# The 5-methylcytosine DNA glycosylase ROS1 prevents paternal genome hypermethylation in Arabidopsis endosperm

**DOI:** 10.1186/s13059-025-03745-w

**Published:** 2025-09-18

**Authors:** Elizabeth A. Hemenway, Mary Gehring

**Affiliations:** 1 Whitehead Institute for Biomedical Research, Cambridge, MA 02142 USA; 2 Department of Biology, Massachusetts Institute of Technology, Cambridge, MA 02139 USA; 3 Department of Biological Engineering, Massachusetts Institute of Technology, Cambridge, MA 02139 USA; 4 Howard Hughes Medical Institute, Whitehead Institute for Biomedical Research, Cambridge, MA 02142 USA

## Abstract

**Background:**

DNA methylation patterning is a consequence of opposing activities of DNA methyltransferases and DNA demethylases. In many plant and animal species, reproduction is a period of significant epigenome lability. In flowering plants, two distinct female gametes, the egg cell and the central cell, are fertilized to produce the embryo and the endosperm of the seed. The endosperm is an unusual tissue, exemplified by triploidy and reduced DNA methylation. In *Arabidopsis thaliana*, a 5-methylcytosine DNA glycosylase, DME, demethylates regions of the central cell genome, leading to methylation differences between maternally- and paternally-inherited endosperm genomes after fertilization. Expression of *DME* in the central cell is required for gene imprinting, or parent-of-origin specific gene expression, in endosperm. *DME* is part of a four member gene family in Arabidopsis that includes *ROS1*, *DML2*, and *DML3*. It is unknown whether any of the other DNA glycosylases are required for endosperm methylation patterning.

**Results:**

Using whole-genome methylation profiling, we identify *ROS1* target regions in the endosperm. We show that *ROS1* prevents hypermethylation of paternally-inherited alleles in the endosperm at regions that lack maternal or paternal allele methylation in wild-type endosperm. Additionally, we demonstrate that at many *ROS1* target regions the maternal alleles are demethylated by DME.

**Conclusions:**

*ROS1* promotes epigenetic symmetry between parental genomes in the endosperm by preventing CG methylation gain on the paternal genome. We conclude that *ROS1* and *DME* act in a parent-of-origin-specific manner at shared endosperm targets, and consider possible implications for the evolution of imprinting mechanisms.

**Supplementary Information:**

The online version contains supplementary material available at 10.1186/s13059-025-03745-w.

## Background

The presence of the modified base 5-methylcytosine (5mC), DNA methylation, is a common feature of eukaryotic genomes. DNA methylation is an epigenetic mark that can be stably inherited through cell divisions and across generations. DNA methylation represses potentially disruptive genomic elements, such as repeats and transposable elements (TEs), and can also contribute to transcriptional regulation of protein coding-genes. Processes that add and maintain 5mC along with those that remove 5mC contribute to genomic DNA methylation patterning. In plants, cytosines are methylated in the CG, CHG, and CHH sequence contexts, where H is an A, T, or C. A family of bifunctional DNA glycosylases/lyases act as DNA demethylases by cleaving the glycosidic bond between the 5mC base and the sugar-phosphate backbone, initiating base-excision repair (BER) [1–3]. Member genes in *Arabidopsis thaliana* are *DME*, *ROS1*, *DML2*, and *DML3*. Homologues of the *DME* family have analogous functions in other flowering plant species [4–7]. Active DNA demethylation opposes RNA-directed DNA methylation (RdDM) [8–10]. RdDM establishes *de novo* DNA methylation in all sequence contexts and maintains a portion of asymmetric CHH methylation [11]. Other pathways also contribute to DNA methylation maintenance. MET1, a homolog of mammalian DNMT1, is recruited to hemi-methylated DNA after DNA replication, and maintains methylation in the CG sequence context by methylating the newly synthesized strand [12, 13]. CHG and some CHH methylation is maintained through positive feedback between the maintenance DNA methyltransferases CMT3/CMT2 and H3K9me2, a histone modification established by SET-domain H3K9 methyltransferases SUVH4/5/6 [14–18].

DNA demethylase targets have been identified in various plant tissues and life stages by whole-genome DNA methylation profiling in plants carrying mutations in DNA demethylases. ROS1 preferentially removes DNA methylation found proximal to the 5′ and 3′ ends of genes, and many of these demethylated regions correspond to the ends of TEs that reside near genes and are targeted by RdDM [8, 10]. *DML2* and *DML3* are more lowly expressed in vegetative tissues and are largely redundant with *ROS1*, but do have unique target sites [8, 19]. *DME* has also been shown to contribute to DNA demethylation in vegetative tissues in a redundant manner with the other demethylases [20] but has primarily been studied for its major function during the reproductive phase of the plant life cycle. *DME* is essential for seed viability [21]. It is active in the central cell and is required for maternal allele demethylation of a subset of TEs, typically short fragments, in the endosperm [3, 6, 22–24]. *DME*/*ROS1* homologs have also been found to be important for seed development in rice and in maize [4, 6, 7, 25]. The role of *DME* in reproduction has been appreciated for some time, but the function of *ROS1*, *DML2*, and *DML3* in plant reproductive tissues has not been extensively investigated. In support of a role for *ROS1* during reproduction, *ROS1* and *DME* have been shown to promote male fertility in Arabidopsis [26, 27]. *ROS1-* and *DME*-mediated DNA demethylation of pollen-specific genes in the vegetative nucleus is required for their expression and promotes proper pollen tube function [27].

The endosperm, a critical seed tissue that supports embryo development, has a unique genetic and epigenetic landscape. Fertilization of the diploid central cell produces the triploid endosperm, which thus has a 2:1 maternal to paternal genome ratio. Endosperm DNA is hypomethylated relative to that in other tissues, in part due to *DME* expression in the central cell of the female gametophyte prior to fertilization, and due to repression of methylation pathways [22–24, 28].

The endosperm is the site of gene imprinting, which refers to biased gene expression from either the maternally-inherited alleles (maternally expressed imprinted genes–MEGs) or the paternally-inherited allele (paternally expressed imprinted genes–PEGs) after fertilization. *DME*-mediated DNA demethylation in the central cell results in maternally-inherited endosperm genomes that are hypomethylated relative to the paternally-inherited endosperm genome; this differential methylation is necessary for gene imprinting [3, 23, 29]. However, biased expression of imprinted genes is not always clearly associated with differential methylation between maternal and paternal genomes in the endosperm, and various epigenetic states have been identified for imprinted genes [30]. PEGs are often characterized by *DME*-dependent differential methylation in their 5′ and 3′ regions, where the repressed maternal allele is demethylated and the expressed paternal allele is methylated, along with H3K27me3, H3K9me2, and CHG methylation accumulation in the gene body of the repressed maternal allele [31–33].

There is prior evidence for a role for *ROS1* in Arabidopsis seeds. *ROS1* promotes expression of the maternally-biased imprinted gene *DOGL4* in the endosperm by preventing full methylation and silencing of the paternal allele [34]. *ROS1* is expressed in dry seeds, and regions dependent on *ROS1* for wild-type methylation levels have been identified in whole dry seeds [35]. *DME*, however, is the only demethylase essential for endosperm development in Arabidopsis. In this study, we investigate the role of *ROS1*, *DML2*, and *DML3* in DNA methylation patterning of the developing endosperm. We find that *ROS1* is required for wild-type DNA methylation patterning in the endosperm, primarily by preventing hypermethylation of the paternal genome. We identify regions in the endosperm that are co-targeted by *DME* and *ROS1*, where *ROS1* promotes a biallelically-demethylated state in wild-type endosperm.

## Results

### *ROS1* contributes to endosperm DNA methylation patterning

**Fig. 1:**
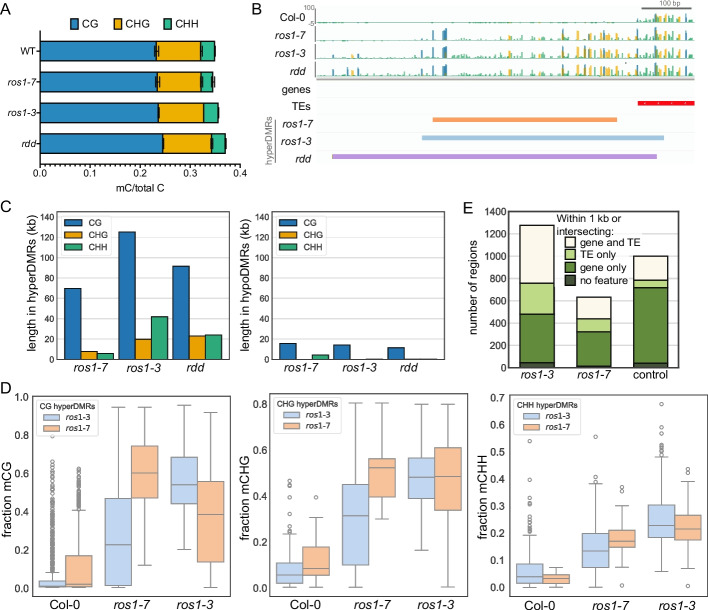
*ROS1* prevents endosperm hypermethylation. **A** Fraction of cytosines by sequence context that are methylated in wild-type Col-0, *ros1-7*, *ros1-3*, and *rdd* in 3 C and 6 C endosperm nuclei. Error bars represent standard deviation from the mean. **B** Example of a region (Chr5, 9,271,400–9,272,200) with DNA hypermethylation in all demethylase mutant backgrounds in the endosperm (blue=mCG, gold=mCHG, green=mCHH). **C** Total length of hyperDMRs (left) and hypoDMRs (right) by sequence context in each mutant. **D** Methylation of *ros1-3* (blue) or *ros1-7* (orange) hyperDMRs in Col-0*, ros1-7* or *ros1-3* endosperm, representing weighted average fraction mC for each *ros1* hyperDMR by sequence context. One biological replicate of each genotype is shown, additional replicates presented in Additional file 1: Fig. S4. Plot is a Tukey’s box plot. **E** The number of *ros1-3* and *ros1-7* hyperDMRs (after merging hyperDMRs identified across sequence contexts into a single list of target regions) found within 1 kb of or intersecting a feature. 1000 randomly selected control regions with low DNA methylation in WT endosperm (<50% in all sequence contexts) were also analyzed

Gene expression data indicate that all four 5-methylcytosine DNA glycosylases are expressed in endosperm [36] (Additional file 1: Fig. S1A). To determine whether 5-methylcytosine DNA glycosylases other than *DME* contribute to the endosperm DNA methylation landscape, we profiled DNA methylation in the absence of *ROS1* and in the absence of *ROS1*, *DML2*, and *DML3*. We performed enzymatic-methyl sequencing (EM-seq) on three replicates each of wild-type Col-0, *ros1*, and *ros1-3 dml2-1 dml3-1 (rdd)* endosperm. For *ros1* profiling, we used both *ros1-3* mutants, which have a T-DNA insertion in exon 7, and *ros1-*7 mutants, which have a missense mutation such that a conserved glutamic acid residue in the DNA glycosylase catalytic domain is changed to a lysine residue [8, 37] (Additional file 1: Fig. S1B). We used two different mutant alleles of *ros1* so we could confirm that observations were not line-specific. Based on previous studies, *ros1*−7 is expected to be a hypomorphic allele [37], whereas *ros1*−3 is a null allele [8]. For all samples, endosperm nuclei were isolated from whole seeds at 7 days after pollination (DAP) by fluorescence-activated nuclei sorting (FANS) based on their triploid DNA content (Additional file 1: Fig. S1C). We obtained methylomes of 98 to 194x genome coverage with high conversion rates (Additional file 2: Table S1). To facilitate direct comparison between endosperm and a vegetative tissue, we also profiled methylation in three replicates each of wild-type Col-0, *ros1-*3, and *ros1-7* rosette leaves, isolating 2 C and 4 C nuclei by FANS (Additional file 1: Fig. S1D, Additional file 2: Table S1). Globally, the total fraction of endosperm methylated cytosines was greater in *rdd* than wild-type in the CG and CHG sequence contexts (Fig. 1A, *p* < 0.05, unpaired *t*-test, Bonferroni-corrected) but was not significantly different between *ros1-3* and wild-type or *ros1-7* and wild-type (Fig. 1A, *p* > 0.05, unpaired *t*-test, Bonferroni-corrected). To identify potential discrete regions of differential methylation we used Dispersion Shrinkage for Sequencing Data (DSS) to identify differentially methylated regions (DMRs) between the demethylase mutant endosperm and the wild type in the CG, CHG, or CHH methylation sequence contexts [38, 39] (Fig. 1B, C). The *ros1-3* mutation is linked to genomic regions from the Ws ecotype on chromosome 2; these regions were removed from analysis in *ros1-3*, *ros1-7*, and Col-0 prior to identifying DMRs to avoid calling false-positive DMRs due to ecotype-specific methylation differences [8, 20]. For identifying DMRs between *rdd* and Col-0, we removed from consideration regions of the Ws genome on chromosomes 2 and 3 that are linked to *ros1-3* and *dml2-1*, respectively [8, 20]. Using DSS, we identified 1,624 total DMRs in any sequence context between *ros1-3* and Col-0, 913 total DMRs between *ros1-7* and Col-0, and 1,319 total DMRs between *rdd* and Col-0 (Additional file 3: Table S2). We partially attribute the lower number of DMRs called in *rdd* relative to *ros1-3* to greater variability between *rdd* biological replicates. Consistent with the molecular function of DNA demethylases, most DMRs were more highly methylated in demethylase mutant endosperm compared to wild-type endosperm; these are referred to as “hyperDMRs” (Fig. 1C, Table 1). The DMRs were short, with the most abundant fraction being 50–100 bp in length (Additional file 1: Fig. S2A). By genome browsing, we observed that DMRs identified by DSS were often surrounded by regions that also appeared differentially-methylated but were not called as DMRs (Fig. 1B). To further investigate, we calculated methylation levels in 50 bp windows 1 kb 5′ and 3′ of hyperDMRs (Additional file 1: Fig. S3). Regions flanking hyperDMRs were more methylated in *ros1* mutant backgrounds than they were in wild-type, up to a few hundred base pairs from the center of the DMR (Additional file 1: Fig. S3).

**Table 1:** Regions of interest identified in this study

**Regions of interest**	**Description**
hyperDMRs	DMRs defined as more methylated in demethylase mutant endosperm relative to wild-type endosperm without respect to maternal or paternal alleles.
*DME* maternal regions	Of DMRs in all sequence contexts identified between maternal and paternal genomes, regions called as maternal hypoDMRs in C24 × Col-0, Col-0 × C24, *ros1-1* × *ros1-3*, and *ros1-3* × *ros1-1*.
*ROS1* paternal, *DME* maternal regions	Of DMRs in all sequence contexts identified between maternal and paternal genomes, regions called as maternal hypoDMRs in both *ros1-1* × *ros1-3* and *ros1-3* × *ros1-1*, and not defined as less methylated on the maternal allele relative to the paternal allele in either C24 × Col-0 or Col-0 × C24.

The fewer *ros1-7* hyperDMRs identified compared to *ros1-3* are consistent with the expectation of *ros1-7* as a hypomorphic allele and *ros1-3* as a null allele (Fig. 1C, Additional file 1: Fig. S1B). To assess the replicability of hypermethylation across the mutant genotypes, we calculated the weighted average of DNA methylation at *ros1*-*3* and *ros1*-*7* hyperDMRs in all genotypes (Fig. 1D, Additional file 1: Fig. S4). Across replicates and genotypes, *ros1* mutants were more highly methylated than Col-0 at hyperDMRs identified in either *ros1* mutant background, indicating a high degree of similarity between the two *ros1* mutant genotypes. Finally, our analysis of methylation across genotypes showed that disruption of *DML2* and *DML3* caused only minor increases in endosperm methylation compared to loss of *ROS1* alone (Fig. 1C, Additional file 1: Fig. S4). Thus, we focused our additional analyses and experiments on *ROS1*.

### *ROS1* prevents DNA methylation spreading from a subset of TEs in the endosperm

**Fig. 2:**
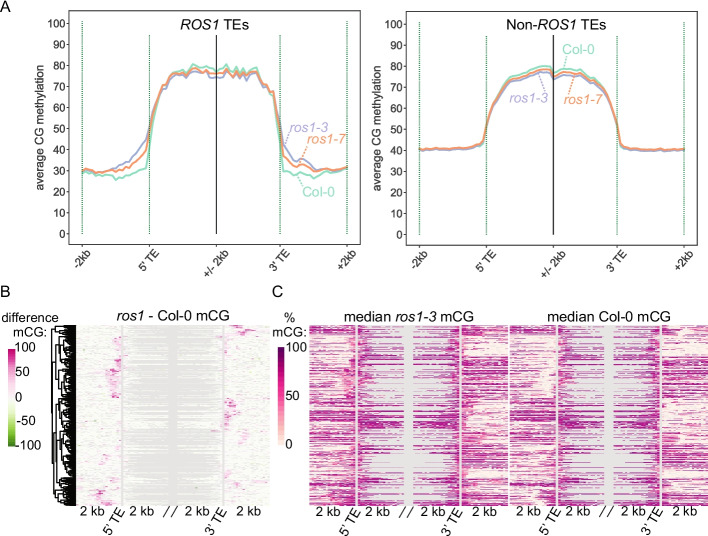
*ROS1* prevents DNA methylation spreading from TEs in the endosperm. **A** Average percent CG methylation determined in 100 bp windows 2 kb outside and 2 kb inside of *ROS1* TEs (left, *n* = 1463). *ROS1* TEs gain DNA methylation at their boundaries in *ros1* mutant endosperm, and are hypomethylated at their boundaries in wild-type endosperm relative to non-*ROS1* TEs (right, *n* = 33279). **B** The difference between median *ros1-3* mCG and median Col-0 mCG value at each 100 bp window 2 kb outside and 2 kb inside of methylated *ROS1* TEs (>10% mC in all sequence contexts in all Col-0 replicates). **C** Underlying data for Fig. 2B, the median *ros1-3* mCG value (left) and the median Col-0 mCG value (right). Gray represents no data. Order of TEs is the same in **B** and **C.**

ROS1 is known to maintain DNA methylation boundaries at TEs, preventing aberrant DNA methylation “spread” [9, 10, 40]. The hypomethylation of the wild-type endosperm genome relative to leaf and seedling tissues prompted us to further investigate the impact of *ROS1* at TE boundaries in endosperm. To identify features of endosperm *ROS1* targets, we first merged *ros1* hyperDMRs (Table 1) from all cytosine sequence contexts. Consistent with previous results, these regions were often found near TEs; over half were within 1 kb of or intersecting a TE (representing 1463 of the 34856 Araport11-annotated TE fragments for *ros1-3*, and 624 for *ros1-7*) (Fig. 1E). For subsequent analyses, we refer to the 1463 TEs within 1 kb of or intersecting a *ros1-3* hyperDMR as *ROS1* TEs (Additional file 4: Table S3). Seventy-nine percent of the DMRs associated with *ROS1* TEs were in the 1 kb flanking regions, rather than in the TE body. In wild-type endosperm, *ROS1* TEs were less methylated in flanking regions (~26% CG methylation) than non-*ROS1* TEs (~40% CG methylation), with a sharper boundary between methylation in the flanking region and in the body of the TE (Fig. 2A, Additional file 1: Fig. S5, 6). Additionally, in *ros1* mutant endosperm, we observed DNA methylation spreading up to ~1 kb beyond the ends of *ROS1* TEs (Fig. 2A). Similar results were obtained for *ROS1* TEs defined using the *ros1-7* methylation data (Additional file 1: Figs. S5, S6). To investigate any endosperm-specific features of *ROS1* TEs, we compared DNA methylation levels of the same TEs in leaves (Additional file 1: Figs. S5, S6). The total level of CG DNA methylation at both *ROS1* TEs and non-*ROS1* TEs was lower in the endosperm than in leaves (Additional file 1: Figs. S5, S6), consistent with endosperm DNA hypomethylation. However, the magnitude of increased CG methylation flanking *ROS1* TEs was not different between endosperm and leaf. We note that bodies of both *ROS1* and non-*ROS1* TEs have higher levels of non-CG methylation in endosperm relative to leaf (Additional file 1: Figs. S5, S6). Overall, we conclude that *ROS1* enforces sharp methylation boundaries at a subset of TEs in the endosperm, as it does in leaves.

Whereas the function of *ROS1* in maintaining DNA methylation boundaries at the ends of TEs has been previously documented, we sought to further characterize the dynamics and mechanism of DNA methylation spreading. To address this, we utilized our high-coverage EM-seq data from Arabidopsis endosperm to investigate the dynamics of DNA methylation spread from individual TEs. We first identified *ROS1* TEs that were methylated in wild-type Col-0 endosperm (those that are at least 10% methylated in all sequence contexts; *n* = 434), as these are TEs from which DNA methylation has the potential to spread. To visualize the molecular phenotype of DNA methylation spreading at individual TEs, we calculated average percent mCG in 100 bp windows of regions flanking TEs for each biological replicate and calculated the difference between the median *ros1-3* replicate and the median Col-0 replicate at each window; differences were visualized using a clustered heatmap (Fig. 2B). We observed that DNA methylation spreading from individual TEs appeared to have a primary direction—either from the 5′ or 3′ end of the TE, which was masked in the meta profile (Fig. 2A). However, by definition, the detection of methylation spreading in *ros1* is only possible if the proximal region of interest has low levels of methylation in wild-type. To test if bidirectional DNA methylation spread from TEs in a *ros1* mutant was possible but did not occur, we separately examined the median wild-type Col-0 value (Fig. 2C, right) and *ros1-3* value (Fig. 2C, left). TEs that were demethylated by ROS1 on both ends were not adjacent to a highly-methylated region on either end in wild-type. In contrast, TEs that appeared to be demethylated by ROS1 on only one end (Fig. 2B) were adjacent to a highly-methylated region on the non-spreading end in wild-type (Fig. 2C). Thus, these TEs lack the capacity for bidirectional spreading in the *ros1* mutant. Therefore, spreading direction reflects the surrounding methylation state in wild-type, rather than being an inherently asymmetric process. One caveat to these analyses is the challenge of TE annotation; incorrect TE boundary annotations could obscure where a true TE “end” is. Future work investigating the nature and mechanism of DNA methylation spreading in a *ros1* mutant will be valuable for understanding the nature and maintenance of epigenetic boundaries in Arabidopsis.

To understand the mechanism of spreading in the endosperm, we examined whether methylation spreading was associated with 24-nucleotide small RNAs, which participate in RdDM, in wild-type Col-0 endosperm [41]. The proximal regions of *ROS1* TEs produced on average more 24-nt sRNAs in wild-type Col-0 endosperm than in the embryo, whereas the opposite was true for non-*ROS1* TEs (Additional file 1: Fig. S7A). However, the data indicated enrichment of endosperm 24-nt sRNAs was driven by only a few *ROS1* TEs (Additional file 1: Fig. S7B). To quantify this, we summed the sRNA levels in 2 kb flanking regions of *ROS1* TEs and identified TEs with a difference between endosperm and embryo that was 1 standard deviation or more from the mean level. This analysis identified 25 TEs driving the observed endosperm enrichment of sRNAs (Additional file 1: Fig. S7B, Additional file 4: Table S3).

To further clarify the relationship between ROS1 activity and endosperm 24-nt sRNAs, we quantified DNA methylation levels in *ros1-3* and wild-type at regions previously determined to be enriched for 24-nt sRNA production in the endosperm relative to the embryo, referred to as endosperm differential sRNA regions (endosperm DSRs) [41]. Endosperm DSRs were lowly methylated in wild-type endosperm, consistent with previous results [41], and a subset gained DNA methylation in *ros1-3* endosperm (Additional file 1: Fig. S7C). To quantify the fraction of endosperm DSRs which are demethylated by ROS1, we calculated a weighted average of CG methylation in endosperm DSRs in Col-0 and *ros1-3*, calculated the difference between median replicates of both, and filtered DSRs which were at least 30% more CG methylated in *ros1-3* than in Col-0. We found 78 DSRs out of 2481 met this stringent threshold. Together, these results suggest that at regions enriched for sRNA production in the endosperm, the low level of DNA methylation observed in wild-type is at least partly a result of ROS1-mediated DNA demethylation, counteracting RdDM.

### *ROS1* targets have reduced capacity for hypermethylation in *ros1* endosperm

**Fig. 3:**
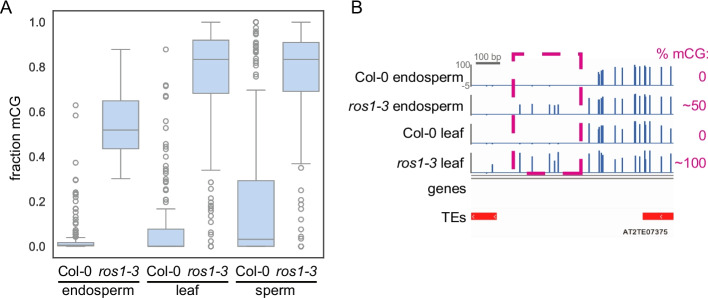
*ROS1* targets display limited hypermethylation in endosperm relative to mutant leaf or sperm. **A** Weighted average fraction mCG in *ros1-3* CG hyperDMRs defined in the endosperm (*n* = 180). Sperm data from Khouider et al. (2021). Plot is a Tukey’s box plot. **B** Genome browsing example of a *ros1-3* CG hyperDMR with limited hypermethylation in *ros1-3* mutant endosperm relative to *ros1-3* mutant leaf (blue=mCG)

To determine whether *ROS1* acts at unique sites in the endosperm, we investigated the extent to which DNA hypermethylation was tissue-specific. We calculated the level of cytosine methylation at endosperm *ros1-3* hyperDMRs (Table 1) in our Col-0 and *ros1-3* leaf methylation data as well as in published data from wild-type Col-0 and *ros1-3* sperm cells [27]. Sperm methylation was of particular interest because the endosperm is a product of fertilization between a sperm and the central cell. In wild-type plant tissues, *ros1-3* CG hyperDMRs displayed DNA methylation features that have been observed at a genome-wide scale: wild-type endosperm has lower DNA methylation levels than does leaf (average 2.8% vs 7.8% in one replicate of each), and sperm has higher DNA methylation levels (~20%) than leaf or endosperm (Fig. 3A, Additional file 1: Fig. S8). Endosperm *ros1-3* hyperDMRs were also hypermethylated in *ros1* mutant leaf and sperm relative to the respective wild-type tissue (Fig. 3A, Supplemental Figures S4 and S8).

However, although *ROS1* endosperm targets are also *ROS1* targets in leaf and sperm, they displayed lower levels of CG DNA methylation in both Col-0 and in *ros1* endosperm (Fig. 3A). What underlies the failure to reach the fully hypermethylated state in *ros1* endosperm? We considered two non-mutually-exclusive possibilities: (1) these regions are variably methylated among nuclei of *ros1* endosperm or (2) these regions are differently methylated between the maternal and paternal genomes in *ros1* endosperm. In support of the second hypothesis, *ROS1* has been shown to prevent hypermethylation of the paternal allele of the *DOGL4* promoter in endosperm (35), and in our data, CG sites in the *DOGL4* promoter were less methylated in *ros1* endosperm compared to *ros1* leaf (Additional file 1: Fig. S9).

### *ROS1* prevents hypermethylation on the paternal genome at target loci

To test if *ROS1* targets are differentially methylated between paternal and maternal genomes we performed allele-specific whole-genome EM-seq using F1 endosperm isolated from reciprocal crosses between *ros1* mutants in the Col-0 (*ros1-3*) and C24 (*ros1-1*) [1] backgrounds, along with appropriate controls. *ros1-1* is a nonsense allele (Additional file 1: Fig. S1B). Endosperm nuclei were collected from three replicates each of Col-0 × C24, C24 × Col-0, *ros1-3* × *ros1-1*, *ros1-1* × *ros1-3*, C24 × C24, and *ros1-1* × *ros1-1* (female parent in cross written first). SNPs between C24 and Col-0 were used to assign reads to a parent-of-origin after sequencing [42] (Additional file 2: Table S1).

**Fig. 4:**
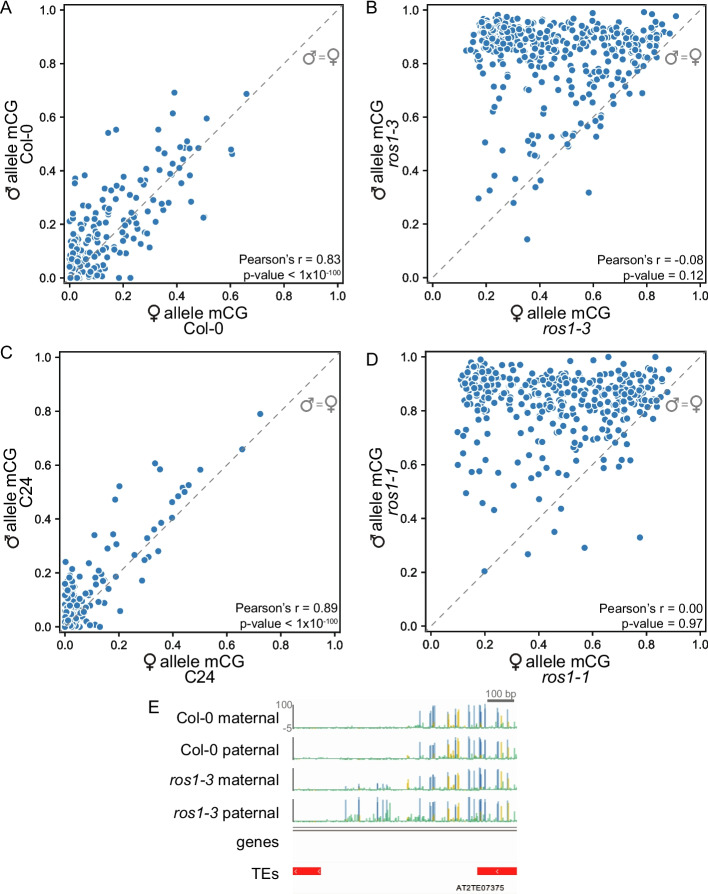
CG hypermethylation in *ros1* mutant endosperm is biased for the paternal allele. Weighted average fraction mCG levels in 418 *ros1-3* CG hyperDMRs, averaged across biological replicates, of maternal and paternal wild-type Col-0 alleles (**A**) and maternal and paternal alleles in *ros1-3* (**B**). Weighted average fraction mCG levels in 346 *ros1-1* CG hyperDMRs, averaged across biological replicates, of maternal and paternal wild-type C24 alleles (**C**) and maternal and paternal alleles in *ros1-1* (**D**). Dashed grey line represents hypothetical perfect correlation between maternal and paternal mCG, not a line of best fit for plotted data. **E** Genome browser example of the region displayed in Figure 3B, now with distinguished maternal and paternal alleles (blue=mCG, gold=mCHG, green=mCHH)

We compared the methylation of maternal and paternal alleles at *ros1-3* hyperDMRs in wild-type and *ros1* endosperm. In wild type, CG methylation of maternal and paternal alleles at *ros1-3* CG hyperDMRs was highly correlated (Pearson’s *r* = 0.83) (Fig. 4A). In contrast, CG methylation of maternal and paternal alleles at the same regions in *ros1* endosperm was not correlated (Pearson’s *r* = −0.08) (Fig. 4B). The lack of correlation was caused by gain of paternal allele methylation in *ros1* endosperm (Fig. 4B). The phenomenon of paternal allele hypermethylation was replicable across ecotypes, as we observed a comparable paternal bias on the C24 genomes at CG hyperDMRs identified between *ros1-1* and wild-type C24 endosperm (Fig. 4C–D, Additional file 2: Table S1,). Consistent with these findings, paternal alleles of *ROS1* TEs gained more mCG in the *ros1-3* mutant than did maternal alleles (Additional file 1: Fig. S10A). Paternal allele hypermethylation of *ros1-3* CG hyperDMRs (defined in Col-0) was also observed when the *ros1-1* allele was inherited paternally (*ros1-3* × *ros1-1*), indicating overlap among regions where *ROS1* prevents paternal hypermethylation in the Col-0 and C24 ecotypes (Additional file 1: Fig. S10B–C).

We also examined parent-of-origin specific methylation for non-CG hyperDMRs. Contrary to CG hyperDMRs, non-CG hyperDMRs were biallelically hypermethylated in their respective sequence contexts (Additional file 1: Fig. S11). Furthermore, non-CG methylation at *ros1-3* CG hyperDMRs was not biased for the paternal allele, although the magnitude of non-CG methylation increase was much less than for CG methylation (Fig. 4E, Additional file 1: Fig. S12). Thus, the observed paternal bias of *ROS1* is specific to the CG sequence context. Studies of ROS1 activity *in vitro* have not revealed a sequence context preference for ROS1 demethylase activity, and *in vivo* whole-genome sequencing data has repeatedly shown that *ROS1* prevents hypermethylation of cytosines in all sequence contexts [2, 8, 43], including the results in the present study of the endosperm (Fig. 1B–D). We propose that it is more likely that the differences observed between sequence contexts indicate *ROS1* target regions are differentially targeted by methylation-establishing and maintenance pathways on the maternal and paternal genomes in the endosperm. Overall, *ROS1* prevents hypermethylation of the paternal allele more strongly than the maternal allele in the endosperm in the CG sequence context.

### *ROS1* promotes a bi-allelically demethylated state in endosperm together with *DME*

Greater paternal genome DNA methylation relative to maternal genome DNA methylation is a distinguishing feature of the endosperm epigenome [22, 23]. It is known that wild-type endosperm maternal allele hypomethylation depends, at least in part, on the activity of *DME* in the central cell before fertilization [3, 6, 22–24]. We have shown that *ROS1* acts to restrict paternal genome hypermethylation in the endosperm. Considering previous work, we hypothesized that in wild-type endosperm the maternal allele of regions that become hypermethylated in *ros1* are demethylated by *DME*. Using previously published data for maternal genome methylation in endosperm where either *dme-2* or wild-type *DME* were inherited maternally [22], we quantified mCG at *ros1-3* CG hyperDMRs and *ros1-7* CG hyperDMRs (Additional file 1: Fig. S13). This analysis showed that the maternal alleles of *ros1-3* and *ros1-7* CG hyperDMRs were hypermethylated in *dme* heterozygous endosperm (Additional file 1: Fig. S13). As expected, the paternally-inherited allele of these regions, which were wild-type L*er* in both cases, were not differentially methylated when *dme* was inherited maternally (Additional file 1: Fig. S13 A, B). This result suggests that a subset of *ROS1* regions are shared demethylase targets, with DME acting on the maternal allele and ROS1 on the paternal allele.

**Fig. 5:**
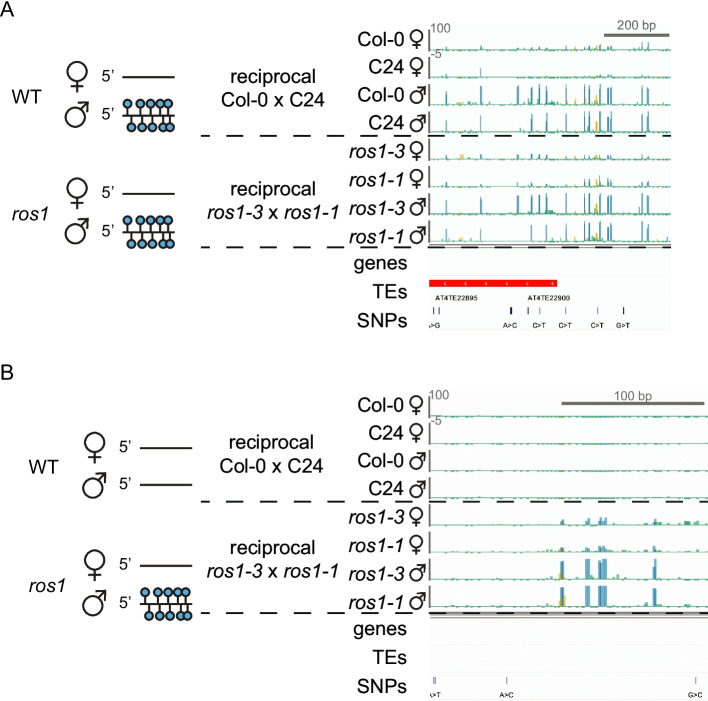
*ROS1* prevents parent-of-origin specific methylation in the endosperm. **A** A diagram and a genome browsing example of a presumed *DME* maternal region in the endosperm (blue=mCG, gold=mCHG, green=mCHH). **B** A diagram and a genome browsing example of a presumed *ROS1* and *DME* shared target in the endosperm, referred to as *ROS1* paternal, *DME* maternal (blue=mCG, gold=mCHG, green=mCHH). Region shown is approximately Chr1:10,173,740-10,173,965

DME activity on the maternally-inherited genome in the central cell of the female gametophyte establishes parent-of-origin-specific DNA methylation in the endosperm. We propose that *ROS1* antagonizes parent-of-origin-specific DNA methylation patterning in the endosperm, resulting in low methylation on both maternal and paternal alleles. To further investigate the role of *ROS1* in the context of greater paternal genome methylation in the endosperm, we used our allele-specific methylation data to identify DMRs between maternal and paternal genomes within a wild-type background and within a *ros1* mutant background (Table 1). For each genotype, DMRs were called in mCG, mCHG, and mCHH sequence contexts independently, but resulting maternally-hypomethylated DMRs were merged into one list of regions per genotype for subsequent analyses. Regions (*n* = 1586) that were demethylated on the maternal allele and methylated on the paternal allele in F1 endosperm of all four reciprocal crosses (WT × WT, *ros1* × r*os1*) were likely regions where *DME* establishes parental DNA methylation asymmetry by acting on the maternally-inherited genome in the central cell before fertilization. This difference is maintained independently of *ROS1*, and we refer to these as “*DME* maternal regions” in the following text and figures (Fig. 5A, Table 1, Additional file 5: Table S4). Regions defined as maternally-hypomethylated in both *ros1* × *ros1* cross directions, but neither WT × WT cross direction were also identified, and based on subsequent analyses we refer to these as “*ROS1* paternal, *DME* maternal regions” in the following text and figures (Fig. 5B, Table 1, Additional file 5: Table S4). These regions (*n* = 274) lacked DNA methylation on both alleles in wild-type endosperm, and gained DNA methylation relative to wild-type predominantly on the paternal allele in the absence of *ROS1*. Hypomethylation of the maternal allele relative to the paternal allele is observable in the *ros1* mutant background at these regions (Fig. 5B). This suggests that in the wild type, the maternal allele of these regions lacks methylation due to a *ROS1*-independent mechanism, whereas the paternal allele is demethylated by *ROS1* (Fig. 5B). The presence of *ROS1* paternal, *DME* maternal regions, in addition to the paternal bias in CG hypermethylation at *ros1* hyperDMRs (Fig. 4), indicates a role for *ROS1* in preventing differential DNA methylation between maternal and paternal genomes in the endosperm, specifically differential methylation where the paternal allele is more highly methylated than the maternal allele.

**Fig. 6:**
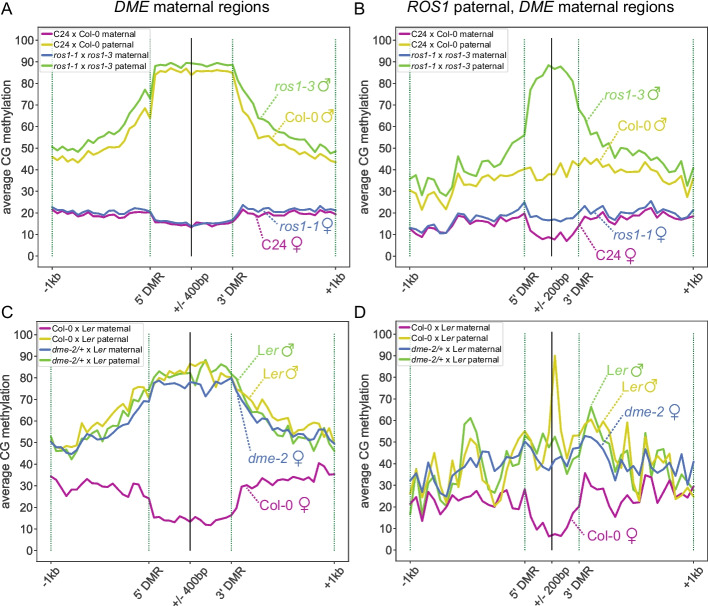
*ROS1* promotes a biallelically demethylated state by preventing hypermethylation of the paternal allele. Percent CG methylation of maternal and paternal genomes from selected biological replicates in *ros1-1* × *ros1-3* and C24 × Col-0 F1 endosperm (reciprocal cross plotted in Additional file 1: Fig. S15) across 50 bp windows, 400 bp inside and 1 kb outside each aligned end of *DME* maternal regions (**A**) and 200 bp inside and 1 kb outside each aligned end of *ROS1* paternal, *DME* maternal regions (**B**). Different distances were evaluated inside the two classes of DMRs to account for the differences in average length (Additional file 1: Fig. S2). Percent CG methylation of maternal and paternal genomes in *dme-2/+* × L*er* and Col-0 × L*er* F1 endosperm across 50 bp windows, 400 bp inside and 1 kb outside each aligned end of *DME* maternal regions (**C**) and 200 bp inside and 1 kb outside each aligned end of *ROS1* paternal, *DME* maternal regions. *dme* methylation data from Ibarra *et al*. (2012) (**D**)

We tested our assumption that maternally-demethylated regions in wild-type endosperm that were not dependent on *ROS1* for proper methylation state (Fig. 5A, “*DME* maternal regions”) were demethylated by DME. Examination of methylation levels in and flanking these regions using published *dme* allele-specific endosperm data [22] indicated maternal allele hypermethylation compared to wild-type, confirming these regions as canonical *DME* targets (Fig. 6A, C, Additional file 1: Fig. S14A). We also examined whether maternal allele hypomethylation at *ROS1* paternal, *DME* maternal regions was, as we hypothesized, dependent on *DME*. We observed maternal allele hypermethylation in *dme* endosperm at these regions but to a lesser extent than at *DME* maternal regions (Fig. 6B, D, Additional file 1: Fig. S14B). We repeated these analyses using an independent, non-allelic *dme* mutant endosperm dataset [24], which further confirmed that *DME* prevents hypermethylation at *DME* and *ROS1* regions (Additional file 1: Fig. S15). Together, these results indicate that *DME*, in part, prevents hypermethylation of the maternal allele and *ROS1* prevents hypermethylation of the paternal allele at some regions that are biallelically-demethylated in wild-type endosperm. Other factors are also likely involved in maternal allele hypomethylation of these regions. For example, it is possible that methyltransferases, such as *MET1*, do not maintain methylation of the maternal allele at these sites in the central cell and early endosperm, which could also prevent full maternal allele methylation irrespective of DNA demethylase activity.

### Relationship between *ROS1* and *DME* in the endosperm

**Fig. 7:**
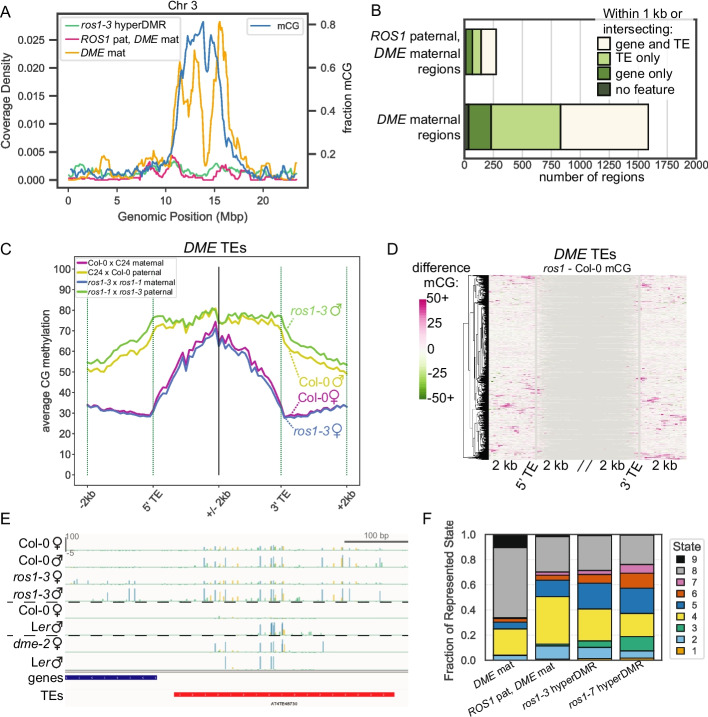
Relationship between *ROS1* and *DME* in the endosperm. **A** Rolling average of DMR coverage density (left *y*-axis) and fraction mCG (right *y*-axis) in 100 kb windows across chromosome 3. **B** The number of *DME* maternal regions and *ROS1* paternal, *DME* maternal regions within 1 kb or intersecting genomic features of interest. **C** Percent CG methylation of Col-0 and *ros1-3* maternal and paternal genomes from selected biological replicates in F1 endosperm across 100 bp windows, 2 kb inside and 2 kb outside each aligned end of TEs within 1 kb or intersecting a *DME* maternal region. **D** The difference between median *ros1-3* mCG and median Col-0 mCG value at each 100 bp window 2 kb outside and 2 kb inside of *DME* TEs. Note that non-allelic data is used for clustered heatmaps. **E** Genome browser example of a TE targeted by DME, where ROS1 prevents DNA methylation spreading from the 5′ end of the TE (blue=mCG, gold=mCHG, green=mCHH). *dme-2* endosperm data from Ibarra *et al*., 2012. **F** The fraction of chromatin states, defined using data from seedling tissue by Sequeira-Mendes *et al*. (2014), represented in endosperm DMRs of each class.

We further investigated the relationship between ROS1 and DME function and targets in the endosperm. To identify the genome neighborhood of *ROS1* and *DME* endosperm targets, we plotted the density of DMRs in 100 kb windows across chromosomes, plotting a rolling average of 10 windows (Fig. 7A). We found that *ROS1* targets, both *ros1-3* hyperDMRs and *ROS1* paternal, *DME* maternal regions, were evenly distributed across chromosomes (Fig. 7A, green and pink lines). *DME* maternal regions, however, were denser in pericentromeric regions (Fig. 7A, gold line). This is consistent with previous results showing that endosperm vs embryo hypoDMRs, indicative of DME activity in the central cell, match the distribution of transposable elements in the genome [23]. In prior work, ROS1 and DME have been associated with distinct genomic features with regards to TEs. ROS1 targets TEs of any length, especially the ends of TEs near gene [8, 10]. In euchromatic regions, DME has been reported to demethylate the bodies of shorter TEs [22–24]. We quantified the number of *DME* maternal regions and *ROS1* paternal, *DME* maternal regions within 1 kb or intersecting a gene or TE (Fig. 7B). More than 86% of *DME* maternal regions and 75% of *ROS1* paternal, *DME* maternal regions were associated with TEs (Fig. 7B).

We observed that the flanking regions of *DME* maternal regions were more highly methylated in wild-type endosperm than were flanking regions of *ROS1* paternal, *DME* maternal regions, most notably on the paternal allele (Fig. 6A, B, compare yellow lines). We also observed a slight increase in CG methylation on the paternal allele at regions flanking *DME* maternal regions in the *ros1* mutant endosperm (Fig. 6A, green *vs*. yellow line). We hypothesized that this may be indicative of a relationship between regions targeted by DME alone and regions targeted by both DME and ROS1. We quantified the distance between each *ROS1* paternal, *DME* maternal region to the nearest *DME* maternal region to test their proximity. The majority of regions were greater than 15 kb away from one another, but about 10% of *ROS1* paternal, *DME* maternal regions were within 1 kb of a *DME* maternal region (Additional file 1: Fig. S16). Given the frequent proximity of both *ROS1* paternal, *DME* maternal regions and *DME* maternal regions to TEs, we investigated if *ROS1* might be preventing DNA methylation spread at the ends of TEs that are demethylated by DME. We identified 3263 TEs that were within 1 kb or intersecting a maternally-demethylated region (*DME* TEs) and calculated allele-specific DNA methylation levels in wild-type and *ros1* (Fig. 7C). As expected, DME-targeted TE bodies and flanking regions were methylated on the paternal allele (50–70% mCG), but the maternal allele was less methylated (~30–60% mCG) in TE bodies and flanking regions (Fig. 7C). A small increase in paternal allele CG methylation at regions flanking *DME* TEs was observed in the *ros1* mutant endosperm (Fig. 7C; green *vs*. yellow lines). To assess how widespread this phenomenon was, we calculated the difference between the median value of mCG in *ros1-3* and Col-0 for *DME* TEs and plotted these using a clustered heatmap (Fig. 7D, Additional file 1: Fig. S17). This visualization shows that *ROS1* prevents methylation spreading at a small subset of *DME* TE ends. To quantify this, we identified 653 TEs that met a threshold of one window or more with at least 30% higher methylation in *ros1-3* than in Col-0. Using this definition, 20% of *DME* targeted-TEs showed some *ROS1*-dependency in their flanking regions in the endosperm. By genome browsing, we observed TEs that were maternally demethylated in a *DME*-dependent manner and where *ROS1* prevented DNA methylation spreading into TE-flanking regions, predominantly on the paternal allele (Fig. 7E).

Although some *DME* maternal regions were proximal to *ROS1* paternal, *DME* maternal regions (Fig. 7C–E), the majority were not (Additional file 1: Fig. S16). Thus we further investigated features that might distinguish *ROS1* and *DME* target regions, examining the overlap with 9 chromatin states defined in seedling tissue [44]. Consistent with the distribution of DMRs (Fig. 7A), we found that in seedling tissue *DME* maternal regions were enriched in H3K9me2-marked heterochromatin in intergenic regions and TEs, with 66% of *DME* maternal regions found in chromatin state 8 (Fig. 7F). The largest single fraction of chromatin states represented in both *ros1-3* and *ros1-7* hyperDMRs was also state 8, but together there was more representation of states 4 and 5 (45.8% for *ros1-3* hyperDMRs), which are marked by H3K27me3 and correspond to upstream regions of promoters and intergenic regions, respectively (Fig. 7F). The largest fraction of *ROS1* paternal, *DME* maternal regions, 48%, were found in chromatin state 4, indicating these shared targets of *ROS1* and *DME* are mostly found in upstream promoter regions marked by H3K27me3 (Fig. 7F). One limitation of this analysis is that it is unknown whether these same chromatin states are present in endosperm tissue. We thus further focused our investigation on H3K27me3 profiles in the endosperm. Using published data from H3K27me3 profiling in Col × L*er* endosperm at 4 days after pollination, we evaluated if *ROS1* and *DME* regions were coincident with H3K27me3 peaks in the endosperm [45]. We found that 32% and 64% of *ros1-3* and *ros1-7* hyperDMRs, respectively, were associated with an H3K27me3 peak in endosperm tissue. By contrast, ~20% of *DME* regions were associated with an endosperm H3K27me3 peak. *ROS1* paternal, *DME* maternal regions were in between these values, with ~29% of regions associated with an H3K27me3 peak in endosperm. These results are consistent with the chromosomal localization and chromatin state of these regions, as H3K27me3 is relatively depleted from pericentromeric regions.

We also investigated *ROS1* and *DME* target regions with regards to imprinting and gene expression in the endosperm. *ROS1* and *DME* have been shown to act together to promote expression of genes in the vegetative nucleus of pollen, ultimately promoting proper pollen germination [27]. In the endosperm, *DME* maternal regions are associated with imprinted genes [31] (Additional file 6: Table S5). Although parent of origin-specific DNA methylation is not an absolute requirement for gene imprinting [30], the relatively demethylated status of both maternal and paternal alleles at *ROS1* paternal, *DME* maternal regions suggests that they are likely not involved in regulation of gene imprinting. Consistent with this notion, no imprinted genes were within 1 kb of or intersecting *ROS1* paternal, *DME* maternal regions. To investigate any differences in expression of DMR-associated genes in the seed, we utilized a single-nucleus RNA sequencing atlas of developing wild-type Arabidopsis seeds [36]. We found that the expression of genes near *DME* maternal regions was enriched in the endosperm compared to the embryo or seed coat, but that the expression of genes near *ROS1* and *ROS1/DME* targets were not enriched in endosperm relative to embryo or seed coat. (Additional file 1: Fig. S18).

We conclude that both ROS1 and DME demethylate TEs, especially those near genes. A subset of TEs demethylated by DME are further demethylated by ROS1 in their flanking regions. However, *ROS1* and *DME* targets are distinguished by their chromosomal distribution and their coincidence with different chromatin states. Furthermore, known imprinted genes are found near *DME* maternal regions, but not near *ROS1* paternal, *DME* maternal regions. Overall, the functional consequence of co-targeting by *DME* and *ROS1*, either on gene expression or chromatin state, is unclear.

### Inheritance of wild-type *ROS1* does not rescue hypermethylation in F1 heterozygous endosperm

How and why does *ROS1* primarily effect paternal allele methylation state in the endosperm? One explanation is that paternal allele-specific demethylation of these regions occurs in the endosperm after fertilization. This would mean that ROS1 selectively acts on paternal alleles despite the presence of maternal alleles. Another, not mutually-exclusive, possibility is that demethylation by ROS1 after fertilization is not allele-specific but that there is no methylation actively established or maintained on the maternal allele to be removed at this point in development. Finally, the paternally-biased effect of *ROS1* in the endosperm could be a product of ROS1 activity pre-fertilization, with the consequent methylation state inherited and maintained after fertilization. Although not expressed in mature pollen or sperm, *ROS1* is expressed in the microspore and bicellular pollen (Additional file 1: Fig. S19) [46, 47] and *ROS1* endosperm targets are hypermethylated in *ros1* sperm (Fig. 3). Thus, *ROS1* could act prior to fertilization in the male germline, leading to inheritance of a demethylated paternal allele in the endosperm, without a requirement for active demethylation of these regions by ROS1 in the developing endosperm after fertilization.

**Fig. 8:**
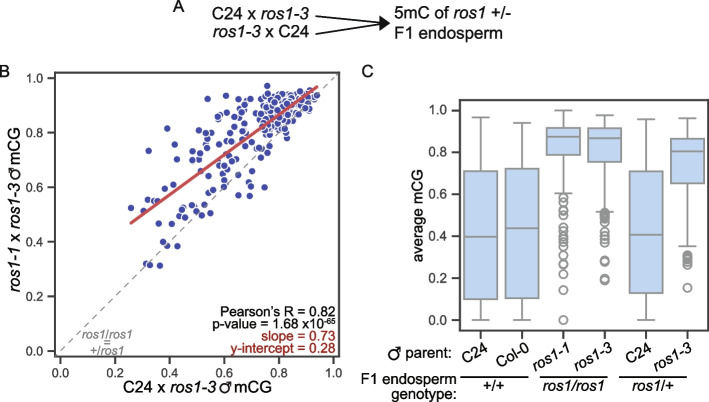
Maternal inheritance of wild-type *ROS1* in the endosperm is not sufficient for complete demethylation of paternal alleles. **A** Graphical depiction of experimental design. To compare genomes inherited from a wild-type *ROS1* background to genomes inherited from a mutant *ros1* background in the endosperm, we reciprocally crossed wild-type C24 and *ros1-3*. F1 endosperm is heterozygous for a wild-type copy of *ROS1*. **B** Weighted average fraction mCG of the paternal allele of *ROS1* paternal, *DME* maternal regions (*n* = 262). Values are averaged across biological replicates of *ros1-1* × *ros1-3* F1 endosperm (*y*-axis) or C24 × *ros1-3* F1 heterozygous endosperm (*x*-axis). Paternal allele methylation was significantly correlated between the homozygous and heterozygous F1 endosperm, where the *ros1-3* allele was inherited paternally. The y-intercept of the line of best fit (red line) indicates that regions are more hypermethylated in the homozygous *ros1* condition than in the heterozygous *ros1* condition. **C** Weighted average fraction mCG of the paternal allele of *ROS1* paternal, *DME* maternal regions (*n* = 262) represented as a Tukey’s box plot, showing gain of paternal allele methylation in *ros1* +/−. Maternally-inherited *ROS1* is not sufficient for complete paternal genome demethylation after fertilization. Additional samples and statistics in Additional file 1: Figs. S20, S21

Based on our comparisons between sperm and endosperm DNA methylation data, we sought to test if inheritance of a wild-type *ROS1* allele is sufficient for paternal genome hypomethylation in the endosperm. More specifically, is the paternal allele hypermethylation that is observed in *ros1* endosperm at *ROS1* paternal, *DME* maternal regions rescued in the presence of a wild-type *ROS1* allele that is inherited maternally? We reasoned that if *ROS1* acts only through the male germline, then a wild-type paternal *ROS1* allele would be necessary for demethylation of the paternal endosperm genome, and a wild-type maternal *ROS1* allele would be insufficient. To test this hypothesis, we performed allele-specific methylation profiling on endosperm of F1 seed derived from reciprocal crosses between *ros1-3* (in the Col-0 background) and wild-type C24 (Fig. 8A). In this design, the F1 endosperm is heterozygous for the *ros1* mutation but either the maternal or paternal sporophytic tissues and female or male gametophytes are null for *ROS1*. If *ROS1* activity is required before fertilization in the paternal sporophyte or male gametophyte to cause hypomethylation of paternal alleles in the endosperm after fertilization, then *ROS1* paternal, *DME* maternal regions will be paternally hypermethylated in heterozygous *ros1-3* endosperm when the mutation is inherited through the paternal parent. In contrast, under this model maternal inheritance of *ros1* should not result in paternal allele hypermethylation in heterozygous endosperm. If *ROS1* instead acts after fertilization to demethylate paternal alleles in endosperm, then paternal allele hypermethylation will not be observed in heterozygous *ros1-3* endosperm when the mutation is inherited through the paternal parent.

We compared the paternal genome average CG methylation level in WT, *ros1*, and *ros1*/+ endosperm at *ROS1* paternal, *DME* maternal regions (Fig. 8B). Paternal allele methylation was correlated between the heterozygous and homozygous *ros1-3* endosperm, when *ros1-3* was inherited paternally (Fig. 8B). We observed that paternal alleles were highly methylated in the C24 × *ros1-3* F1 heterozygous endosperm, like in *ros1* endosperm, relative to wild-type paternal alleles (Fig. 8C, Additional file 1: Fig. S20, “*ros1-3* paternal”). Thus, inheritance of a wild-type maternal *ROS1* allele is not sufficient for wild-type mCG levels at *ROS1* paternal, *DME* maternal regions. We also observed little difference in methylation on maternally-inherited alleles between heterozygous and homozygous *ros1* mutants relative to wild-type; maternal allele methylation was low in these regions regardless of the *ROS1* genotype (Additional file 1: Fig. S20 “*ros1-3* maternal”). We conclude that the majority of paternal allele hypermethylation in *ros1* mutant endosperm is inherited through the male germline. Thus, in wild-type, the majority of regions are demethylated by ROS1 prior to fertilization. However, we observed a slight, non-significant, reduction in paternal mCG at *ROS1* and *DME* regions in the *ros1*/+ F1 heterozygous endosperm relative to the *ros1* homozygous endosperm (Fig. 8B–C, Additional file 1: Fig. S20 “*ros1-3* paternal”) indicative of active maternal *ROS1* in the endosperm after fertilization that is able to partially rescue paternal CG hypermethylation. However, in the same C24 × *ros1-3* F1 heterozygous endosperm, methylation of maternal alleles (inherited from C24) of *ROS1* paternal*, DME* maternal regions was also decreased (Additional file 1: Fig. S20, “C24 maternal”), so this reduction is likely not indicative of specific ROS1 activity on paternal alleles only in the endosperm post-fertilization.

We also investigated the role of maternally and paternally-inherited *ROS1* in the endosperm at the larger set of *ros1-3* CG hyperDMRs. Paternal allele methylation was also correlated between C24 × *ros1-3* F1 heterozygous endosperm and *ros1-1* × *ros1-3* F1 endosperm at *ros1-3* CG hyperDMRs (Additional file 1: Fig. S21). Maternally-inherited wild-type *ROS1* was not sufficient for wild-type methylation levels on paternal alleles of *ros1-3* CG hyperDMRs (Additional file 1: Fig. S21B, “*ros1-3* paternal”). We again observed a slight decrease in paternal allele hypermethylation at some *ros1-3* CG hyperDMRs in the *ros1*/+ F1 heterozygous endosperm (Additional file 1: Fig. S21A, 21B “*ros1-3* paternal”). Additionally, we observed a non-significant increase in paternal allele mCG in the *ros1*/+ F1 heterozygous endosperm when *ros1-3* was inherited maternally (Additional file 1: Fig. S21B, “C24 paternal”), further implicating maternally-inherited *ROS1* in preventing hypermethylation at a subset of *ROS1* target regions in the endosperm after fertilization. Overall, we conclude that paternal allele CG methylation patterning at *ROS1* regions is largely inherited through the male germline, but some *ROS1* regions are actively demethylated by ROS1 in the endosperm on both maternal and paternal alleles, post-fertilization.

Finally, as it has been shown that ROS1 and DME act together at some regions in pollen [27], we investigated whether *DME* had any role in the male germline at *ROS1* target regions. As expected, *ROS1* paternal, *DME* maternal regions were hypermethylated in *ros1-3* mutant sperm relative to wild-type sperm (Additional file 1: Fig. S22A). In sperm collected from *dme-2* heterozygous plants, *ROS1* paternal, *DME* maternal regions were not hypermethylated (Additional file 1: Fig. S22A), although sufficient sperm methylation data was only available for 103 regions. To evaluate more *ROS1* target regions, we performed the same analysis using *ros1-3* and *ros1-7* CG hyperDMRs. There was a significant increase in CG methylation at *ros1-3* and *ros1-7* CG hyperDMRs in *dme-2* heterozygous sperm compared to wild-type, although of a lower magnitude than that observed in *ros1* sperm, suggesting DME is partially capable of demethylating ROS1 target regions earlier in development, which could further impact paternal allele methylation in the endosperm (Additional file 1: Fig. S22B–C). Overall, these results are consistent with previous reports that *DME* contributes to demethylation in somatic tissues and in the pollen vegetative nucleus, but not to the same extent as *ROS1* [20, 48].

## Discussion

Loss of DNA demethylase activity in Arabidopsis is well-tolerated under normal laboratory growth conditions, except when it occurs in the female lineage that gives rise to endosperm. The endosperm is uniquely sensitive to epigenome disturbances, making it an informative tissue in which to study DNA methylation patterning and mechanisms. Studies of the DNA demethylase-encoding gene, *DME*, highlight this point—*DME* is required in the female gametophyte for proper seed development. DME activity before fertilization establishes an asymmetric DNA methylation state between the maternal and paternal genomes in endosperm, where maternally-inherited alleles are hypomethylated [3, 21, 49]. Although *ROS1* is not essential for seed development in Arabidopsis, we have shown that *ROS1* is also required to maintain proper DNA methylation patterning in the endosperm, although it acts at far fewer regions than *DME*. We first identified *ROS1* target regions in the endosperm using high-quality, high-coverage whole genome DNA methylomes generated by enzymatic-methyl sequencing. These regions were also demethylated by ROS1 in leaf tissue. However, in *ros1* endosperm, methylation gain was biased for the paternal genome. Thus, *ROS1* prevents paternal genome hypermethylation in the endosperm.

**Fig. 9:**
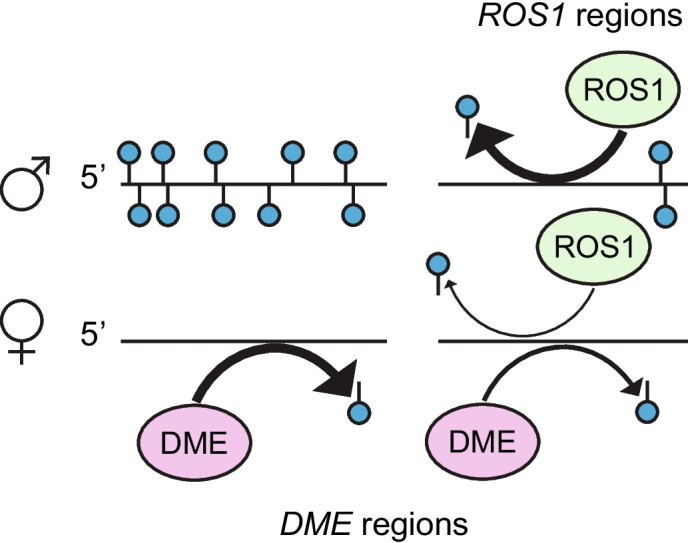
A model for CG methylation patterning in endosperm at *ROS1* targets. *ROS1* prevents endosperm hypermethylation of the paternal allele, and to a lesser extent the maternal allele, at *ROS1* regions (representing both *ros1* hyperDMRs and *ROS1* paternal, *DME* maternal regions), indicated by relative arrow thickness. *DME* prevents endosperm hypermethylation of the maternal allele at *DME* regions, a subset of which depend on *ROS1* for paternal allele demethylation. In this study, low levels of paternal allele DNA methylation in the endosperm at *ROS1* regions primarily depend on paternally-acting *ROS1* prior to fertilization, analogous to maternally-acting *DME* in the central cell

The relationship between *DME* and *ROS1* is intriguing, given the role of *DME* in demethylating maternally-inherited endosperm genomes and our discovery of a function for *ROS1* in demethylating the paternally-inherited endosperm genome. We identified canonical *DME* target regions (those with hypomethylated maternal alleles and a methylated paternal allele) using our wild-type endosperm data and previously published *dme* mutant endosperm data [22, 24] (Figs. 5, 6, and 7). At these regions, methylation status on both maternal and paternal alleles is largely *ROS1*-independent, although there is a slight gain in paternal allele CG methylation in *ros1*, particularly at the ends of regions exhibiting *DME*-dependent maternal hypomethylation (Figs. 6A, 7C–E). We identified a newly-described class of regions that lack methylation on both alleles in wild-type endosperm, but gain methylation in a *ros1* mutant, primarily on the paternal allele. We showed that these regions are partly dependent on *DME* for lack of methylation on the maternal allele in wild-type endosperm, whereas *ROS1* prevents paternal allele methylation (Figs. 6, 9). The methylation features of these regions differ from canonical *DME* regions. Methylation gain on the maternal allele in *dme* endosperm does not reach the same level as the methylation on paternal alleles in *ros1* endosperm or the level of methylation of maternal alleles of canonical DME targets in *dme* endosperm (Fig. 6). Low maternal allele methylation at these regions could also be a result of lack of methyltransferase targeting, an interesting direction for future studies. In summary, *ROS1* parent-of-origin specific targets are also *DME* targets, but *DME* parent-of-origin specific targets are not *ROS1* targets (Fig. 9).

DME is thought to act largely before fertilization in the central cell to create parent-of-origin specific methylation patterns in the endosperm, where maternal alleles are hypomethylated compared to paternal alleles at canonical targets. Similarly, paternal allele hypermethylation in *ros1* endosperm might be caused by the action of *ROS1* before fertilization. In support of this idea, we found that the paternal hypermethylation of *ROS1* target regions in *ros1* endosperm is largely inherited through the male germline (Fig. 8, Additional file 1: Fig. S20, 21). Inheritance of a wild-type maternal *ROS1* allele in endosperm is not sufficient for removal of methylation from paternal alleles, but contributes to low levels of DNA methylation on both maternal and paternal alleles in wild-type endosperm post-fertilization (Additional file 1: Fig. S20, 21). It is presently unknown in what tissues or cells ROS1 acts before fertilization to achieve paternal allele demethylation. *ROS1* is not known to be expressed in sperm, but is expressed earlier during microsporogenesis (Additional file 1: Fig. S19).

It has not escaped our notice that in *ros1* endosperm the *ROS1* paternal, *DME* maternal regions resemble canonical *DME* regions associated with imprinted genes in wild-type endosperm, with a methylated paternal allele and a hypomethylated maternal allele (Figs. 6, 9). This observation suggests a mechanism whereby new parent-of-origin specific asymmetric methylation patterning might arise, potentially leading to the gain of gene imprinting. We have shown that *ROS1*, together with *DME*, prevents parent-of-origin specific methylation patterning in endosperm (Fig. 9). For a region to gain the potential for imprinting, it might need to both lose *ROS1* target status in the male germline but retain *DME* target status in the female gametophyte. Future work will investigate the molecular and genetic relationship between *ROS1* and *DME* in reproductive tissues to further our understanding of gene imprinting mechanisms in the seed.

## Conclusions

We have performed an in-depth characterization of the effect of the DNA demethylase-encoding gene *ROS1* on DNA methylation patterning in the *Arabidopsis thaliana* endosperm. Our work shows that *ROS1* prevents CG hypermethylation at discrete regions of the endosperm genome, with a strong bias for the paternal allele. We show that the maternal allele demethylation of *ROS1* targets in wild type is in part dependent on *DME.* Thus, proper patterning of the endosperm epigenome depends on a *ROS1* effect on paternal alleles and a *DME* effect on maternal alleles.

## Methods

### Plant material

Plants for non-allele-specific methylation profiling in endosperm and leaf (Figs. 1, 2, and 3) were grown in a Conviron growth chamber at 22 °C using 22 h light/2 h dark cycles with 120-µm fluorescent light. Plants for allele-specific methylation profiling (Figs. 4, 5, 6, and 7) were grown using 16 h light/8 h dark cycles; set-point for temperature was 22 °C. The mutants *ros1-3* and *ros1-3*; *dml2-1*; *dml3-1* were originally described in Penterman et al. [8]. The *ros1-7* allele was described in Williams et al. [37]. In the C24 background, the *ros1-1* mutant allele was described in Gong et al. [1] and was obtained from ABRC (CS66099) [1]. Genotypes for all plants were PCR-verified*.*

### Preparing endosperm samples for EM-seq

Flowers were emasculated and pollinations were performed 2 days after emasculation. Seven days after pollination (DAP), seeds were removed from 3 to 6 siliques and placed into 120 μl of nuclei extraction buffer, available as part of the CyStain UV Precise P kit (Partec North America #055002) on ice. Seeds were ground manually in microfuge tubes using a sterile blue pestle. 800 μl of companion Nuclei Staining Buffer was added to ground seeds, and the mixture was filtered 1–2 times through sterile Cell Trics 30 μm filters and kept on ice. 3 C and 6 C nuclei were then sorted into 200 μl of Buffer ATL from Qiagen QIAamp DNA micro kit (#56304) with BD FACSAria SORP equipped with a 100 mW UV laser using a nozzle size = 100 µm, Sheath pressure = 20 psi. 20 μl of Proteinase K solution was added to the nuclei mixture and incubated overnight at 56 °C. The next day, remaining steps for QIAamp DNA micro kit were performed, following the protocol for “Isolation of Genomic DNA from Tissues.” DNA was eluted in 20 μl of nuclease-free water and kept at −20 °C until all samples were prepared.

### Preparing leaf samples for EM-seq

Rosette leaf tissue from 4-week-old plants was collected and chopped manually in nuclei extraction buffer using a razor blade. All following nuclei preparation steps were the same as for seeds. For leaf tissue, 2 C and 4 C nuclei were collected using the BD FACSAria conditions described above. DNA extraction steps were the same as for endosperm nuclei.

### EM-seq library prep

To prepare converted libraries for whole-genome sequencing of DNA methylation, we used the EM-seq kit from New England Biolabs (#E7120S). 1 μl diluted 1:100 pUC19 control DNA per 10–30 ng sample DNA was spiked in as per kit instructions. Using a Covaris sonicator, DNA samples for the non-allelic seed and leaf were sheared in 130 μl of 1X TE buffer, 6 × 16 mm tubes using conditions: [Peak power = 175 W, Duty factor = 10% Cycles/burst = 200, Time = 180 sec]. TE buffer was then exchanged for 50 μl nuclease-free water using a 1X Ampure bead clean up before continuing the EM-seq protocol. Conditions were later optimized, and for the allelic endosperm experiments DNA was sheared in 50 μl of 1X TE buffer, 6 × 16 mm tubes under conditions [Peak power = 175 W, Duty factor = 10%, Cycles/burst = 200, Time = 80 sec] with no subsequent buffer exchange. All remaining library prep steps were then performed as written in the EM-seq manual; we used 7–8 cycles of PCR amplification to amplify libraries. Libraries were pooled at approximately equimolar concentration using library concentrations quantified from Fragment Analyzer readouts prior to a final bead clean-up of remaining adapter fragments, and sequenced using Illumina NovaSeq S4, 120 × 120 paired end reads.

### Processing non-allele specific EM-seq data

#### Data processing was performed based on the Bismark pipeline

(https://felixkrueger.github.io/Bismark/) [50]. Briefly, reads were trimmed and filtered using Trim galore using standard settings for phred33 quality control and paired-end Illumina reads (https://github.com/FelixKreger/TrimGalore). Trimmed reads were mapped using Bismark alignment to the TAIR10 Arabidopsis genome containing the pUC19 methylated control sequence. The number of mismatches allowed in a seed alignment (--N) was set to 1 and the maximum insert size (--X) to 600. Bismark deduplicate was used to remove PCR duplicates using default settings. Methylation information was extracted from deduplicated reads with Bismark Methylation Extractor using default settings for paired-end reads. Initial conversion and protection quality checks, and analysis of whole-genome methylation status were performed (Additional file 2: Table S1). Where applicable, we excluded the region of annotated Ws homology, Chr2: 8,802,496–15,397,296, from *ros1-3* endosperm and leaf samples and the matched wild-types prior to DMR calling [20]. We removed the same region of Chr 2 as well as the annotated Ws region at Chr3: 688,340–5,117,803 from *rdd* endosperm and matched wild-types prior to DMR calling [20]. The presence or absence of Ws regions did not have an out-sized effect on DMR identification, and we did not remove Ws regions from *ros1-3* data in the allelic analysis for DMR calling.

### C24 pseudogenome generation

To facilitate mapping reads from the C24 ecotype in F1 hybrids, we generated a TAIR10 genome with substituted SNPs for the C24 variant [42]. We then prepared this genome for mapping using Bismark genome preparation with default settings.

### Processing allele-specific EM-seq data

Reads were trimmed and filtered using Trim galore using standard settings for phred33 quality control and paired-end Illumina reads. Trimmed reads were then mapped to TAIR10 genome (containing the pUC19 methylated control sequence when applicable) using Bismark alignment. The number of mismatches allowed in a seed alignment (--N) was set to 1 and the maximum insert size (--X) to 600. Bismark deduplicate was used to remove PCR duplicates using default settings. Methylation information was extracted from deduplicated reads using Bismark Methylation Extractor using default settings for paired-end reads. Initial conversion and protection quality checks and analysis of whole-genome methylation status were performed at this stage. Remaining reads that did not map to TAIR10 were then mapped to the pseudo-C24 genome and deduplicated. Mapped reads from both mapping runs were assigned to a parent-of-origin using the SNPs identified between Col-0 and C24 by Jiao et al. (2020), accounting for C-T or G-A SNPs, using the script assign_to_allele.py [51]. Methylation information was then extracted from each parental genome individually using Bismark Methylation Extractor, before combining outputs from the same parental genomes from the two mapping runs. The methylation data from Ibarra et al. (2012) [22] was processed and mapped using the same pipeline, but with modifications for single-end data in Bismark, and a TAIR10-L*er* pseudogenome and SNPs were used.

### Identifying differentially methylated regions (DMRs)

DMRs were called using Dispersion Shrinkage for Sequencing data (https://rdrr.io/bioc/DSS/) [38, 39]. A cytosine was required to have 3 supporting reads to be included in the DSS analysis. The following parameters were utilized: [DMR.delta=0.3 (CG), 0.2 (CHG), 0.1 (CHH); DMR.p.threshold = 1e-02; DMR.minlen = 50; DMR.minCG = 5; DMR.distance.merge = 100; DMR.pct.sig = 0.1].

### Merging DMRs

When applicable, we compiled DMRs for each mutant genotype identified in the CG, CHG, and CHH sequence contexts. We used Bedtools merge to merge any overlapping DMRs (which would occur if a region was called as a DMR in more than one sequence context) into one target region.

### Summing methylation across regions of interest

For summed mC analyses, as in Figs. 1D, 3A, 4A–D, 8B–C, input methylation data for cytosines with at least 5 informative reads were intersected with regions of interest using Bedtools intersect. Then, a weighted mean methylation level was calculated for each region, weighted by sequencing depth at each cytosine [31]. For Tukey’s box plots, only regions of a given category with data in all plotted samples and replicates were plotted unless specified. For plotting on a scatterplot (Figs. 4A–D, 8B), the average value across biological replicates was plotted, and only regions with data in all replicates were plotted. When reported, *t*-tests were performed between the average of weighted means in biological replicates using Scipy stats (https://docs.scipy.org/doc/scipy/reference/generated/scipy.stats.ttest_ind.html).

### Analysis of average methylation and 24-nt sRNA patterns for features of interest

For each feature, average percent mC value was calculated over set windows inside and outside the feature. Window lengths and length surrounding feature ends are described for each analysis in the results. An average was then calculated for each window across the meta-feature and plotted (Figs. 2 A, 6, 7C) [51]. When performing this analysis using 24 nt sRNA data (Additional file 1: Fig. S7A), an average value of coverage per base pair was used as input data [41]. For clustered heatmaps (Figs. 2B, C, 7D, Additional file 1: Fig. S17), the window calculations for each feature were plotted; if replicates were available the median replicate value at each window was plotted. Clustered heatmaps were generated using R package pheatmaps (https://www.rdocumentation.org/packages/pheatmap/versions/1.0.12/topics/pheatmap). Rows (features of interest) were clustered using hierarchical agglomerative clustering, calculating Euclidean distance measurements between rows. To identify TEs driving the endosperm enrichment of 24nt sRNAs, reads per base (RBP) were summed across calculated windows in either flanking region of each TE, before filtering for TEs with a summed value in either flanking region greater than 1 standard deviation from the mean value across all TEs.

### Calculating DMR density across chromosomes

One hundred kb windows were defined across the TAIR10 genome assembly, then DMR density was calculated in each window using Bedtools coverage. The resulting density values were then plotted as a centered rolling average over 10 windows across individual chromosomes (Fig. 7A). The methylation values on these plots represent CG methylation in one Col-0 endosperm replicate, averaged across the same windows using the summed methylation approach described above, and a centered rolling average over 10 windows of these data was plotted on the same *x*-axis.

## Supplementary Information

Additional file 1: Figures S1–S22.

Additional file 2: Table S1: Methylation mapping statistics and conversion rates.

Additional file 3: Table S2: DMRs identified in endosperm.

Additional file 4: Table S3: TEs within 1 kb or intersecting an endosperm *ros1-3* *vs*. Col-0 hyperDMR.

Additional file 5: Table S4: List of *DME* maternal regions and *ROS1* paternal, *DME* maternal regions.

Additional file 6: Table S5: Imprinted genes within 1 kb or intersecting a DMR.

Additional file 7: Review history.

## Data Availability

EM-seq data generated in this study is available through the Gene Expression Omnibus database under accession number GSE280598 (https://www.ncbi.nlm.nih.gov/geo/query/acc.cgi?acc=GSE280598) [60]. Additional scripts used to process and analyze data are available on Github (https://github.com/Gehring-Lab/ROS1_endo) under an MIT license for usage and archived using Zenodo [61, 62]. Sources of published code used for analysis are referenced in the methods as applicable. Published datasets available through GEO GSE15922 [63], GSE94792 [56], GSE141154 [57], GSE38935 [58], GSE295007 [59], were used and referenced accordingly.
