# Supplementary material for: The 5-methylcytosine DNA glycosylase ROS1 prevents paternal genome hypermethylation in Arabidopsis endosperm
Source: Genome Biol. 2025 Sep 18;26:286. doi: 10.1186/s13059-025-03745-w (PMC12445040; doi:10.1186/s13059-025-03745-w)
Supplement: Supplementary file 1 — Additional file 1: Figures S1–S22. [file 13059_2025_3745_MOESM1_ESM.pdf]

Additional file 1
Hemenway and Gehring, 2025

The 5-methylcytosine DNA glycosylase ROS1 prevents paternal genome hypermethylation in Arabidopsis endosperm

Figures S1-S22

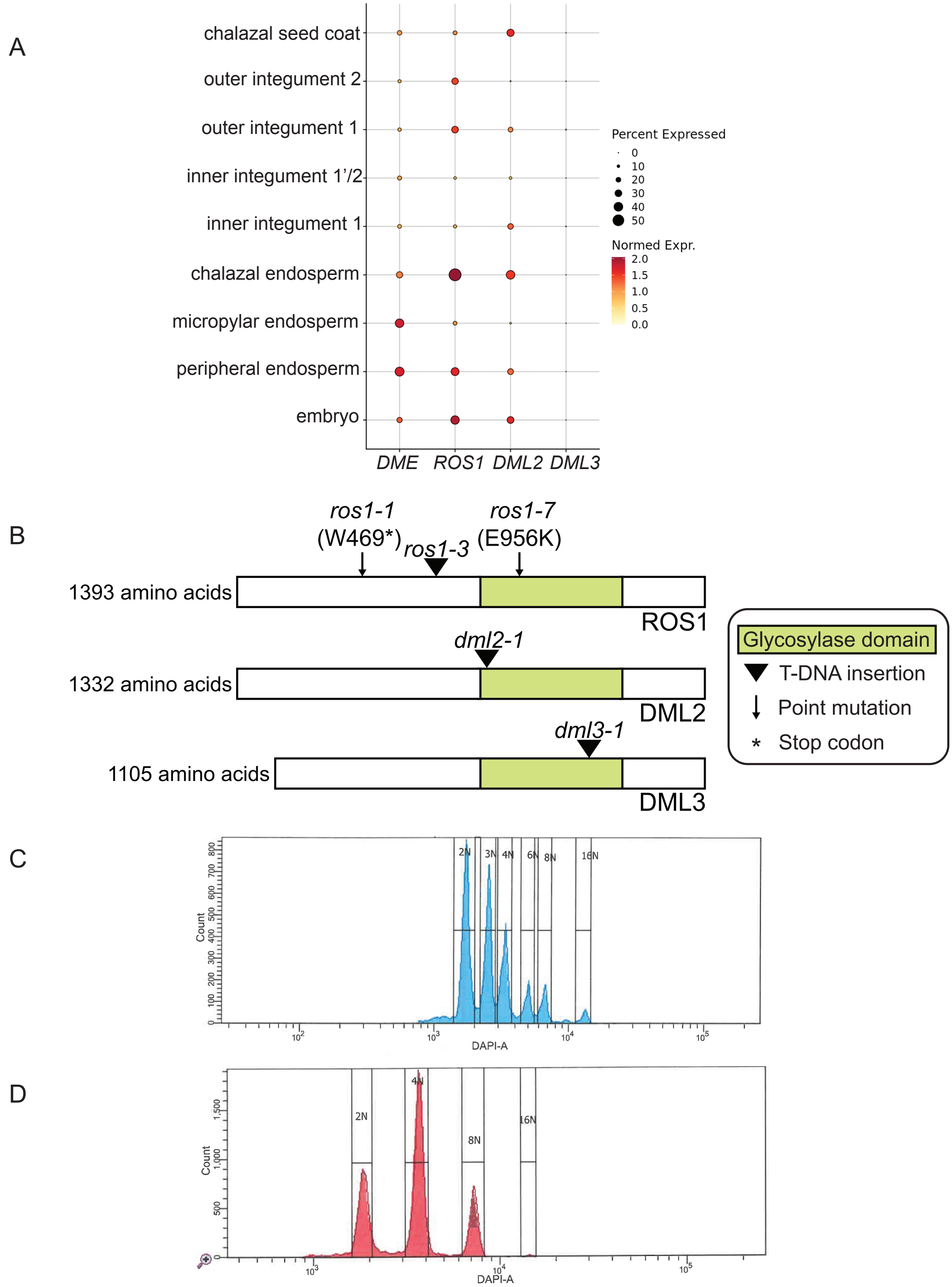

Fig. S1: Demethylase expression in seeds, mutant alleles, and flow cytometry profiles of seed and leaf. A) Expression of *DML* family genes in seed tissues at 7 days after pollination. Single-nucleus mRNA-seq data is from Martin *et al.*, 2025. Size of circle is percent of nuclei of that type in which the transcript is detected; color represents the normalized expression level of the gene. B) DNA demethylase mutant alleles used in this study. C) Representative FANS plot for 7 DAP seeds sorted by DAPI fluorescence. C) Representative FANS plot for rosette leaf tissue sorted by DAPI fluorescence.

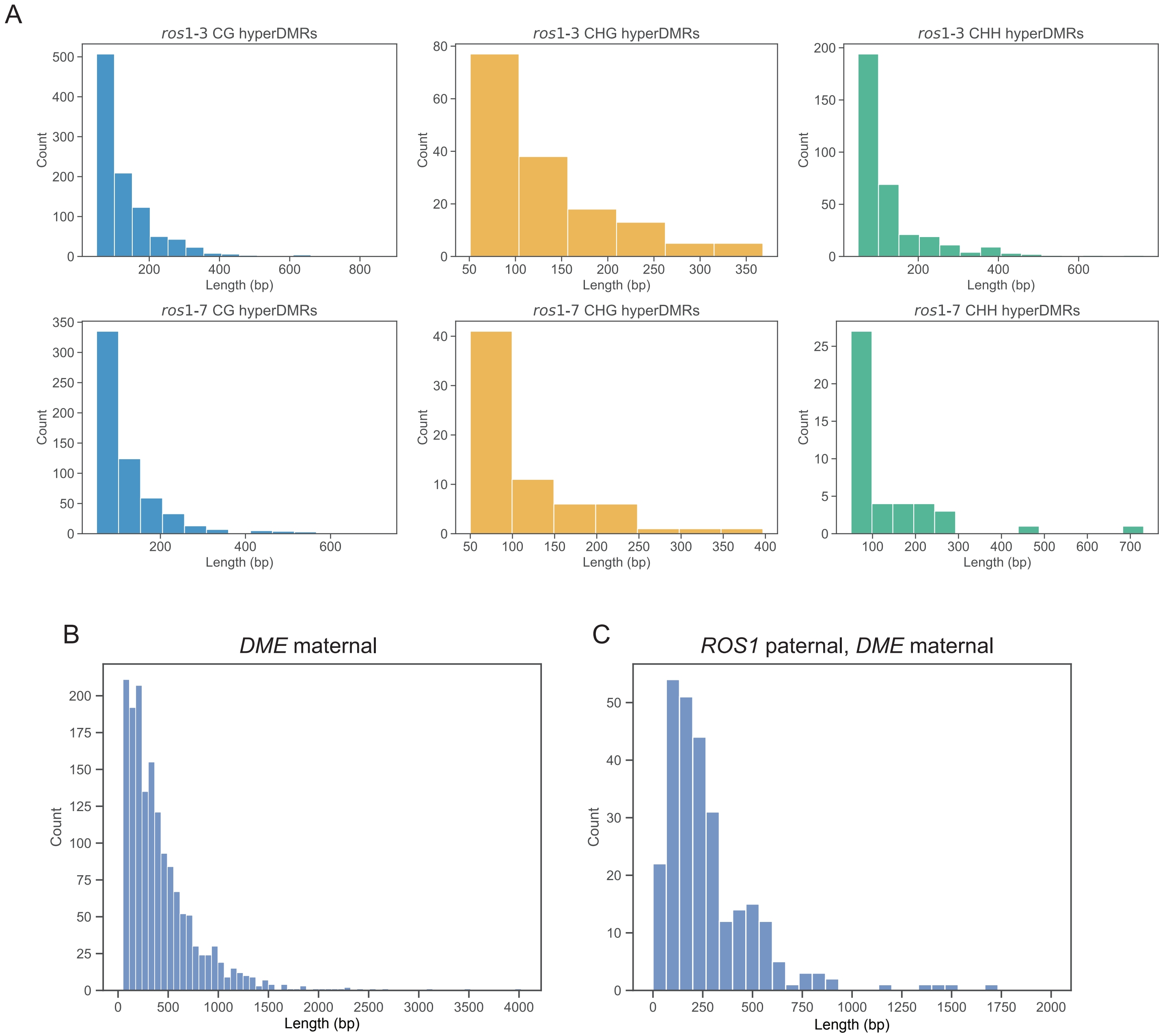

Fig. S2: Length of Differentially Methylated Regions (DMRs). A) Length of hyperDMRs called by DSS between *ros1* mutant endosperm and wild-type Col-0 endosperm. B) Length distribution of *DME* maternal regions. C) Length distribution of *ROS1* paternal, *DME* maternal regions.

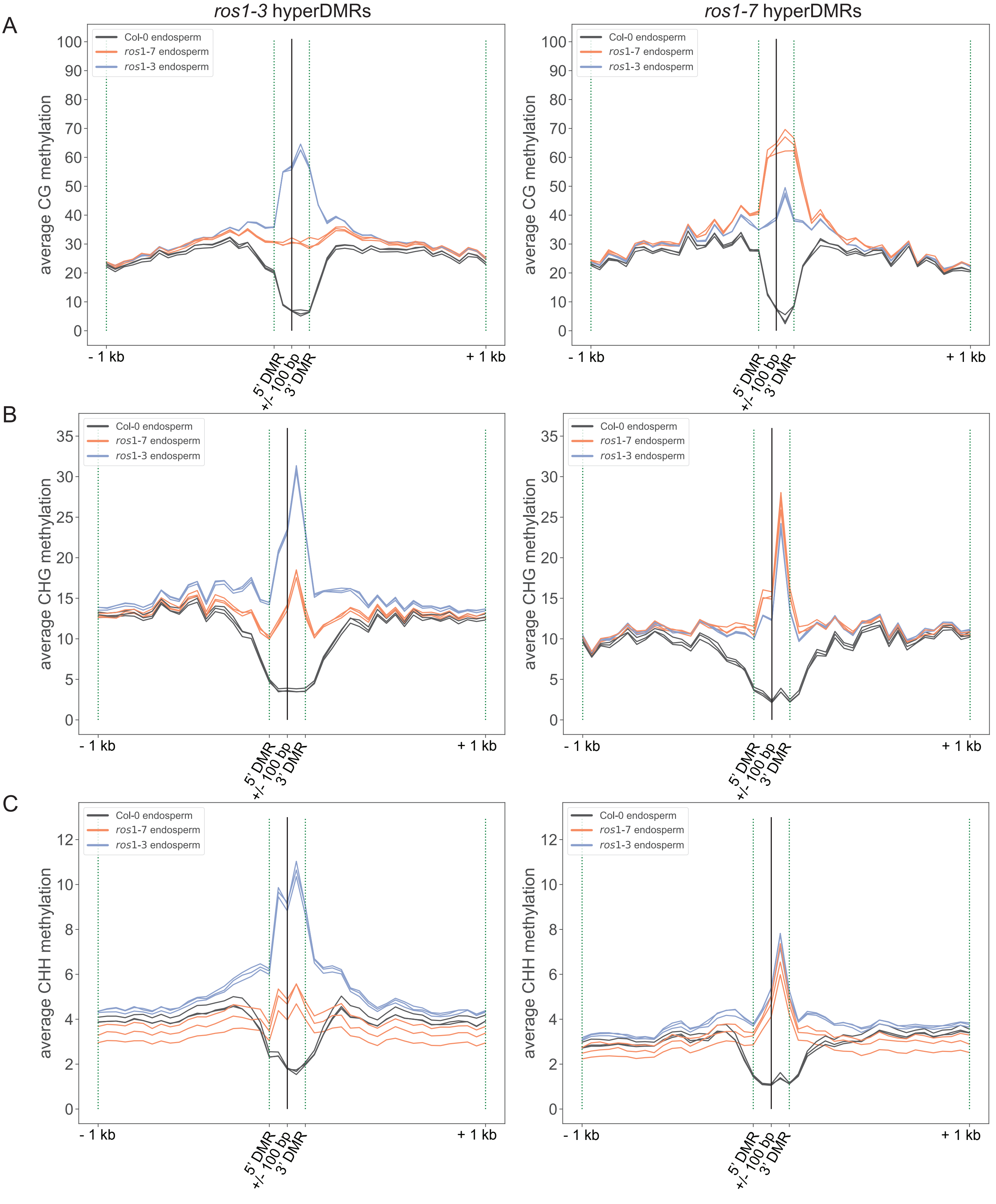

Fig. S3: *ros1* hyperDMRs display additional hypermethylation in proximal regions. Average percent DNA methylation determined in 50 bp windows 1 kb outside and 100 bp inside of *ros1-3* hyperDMRs (left) and *ros1-7* hyperDMRs (right) called in the A) CG, B) CHG, and C) CHH sequence contexts. Biological replicates are plotted in the same color.

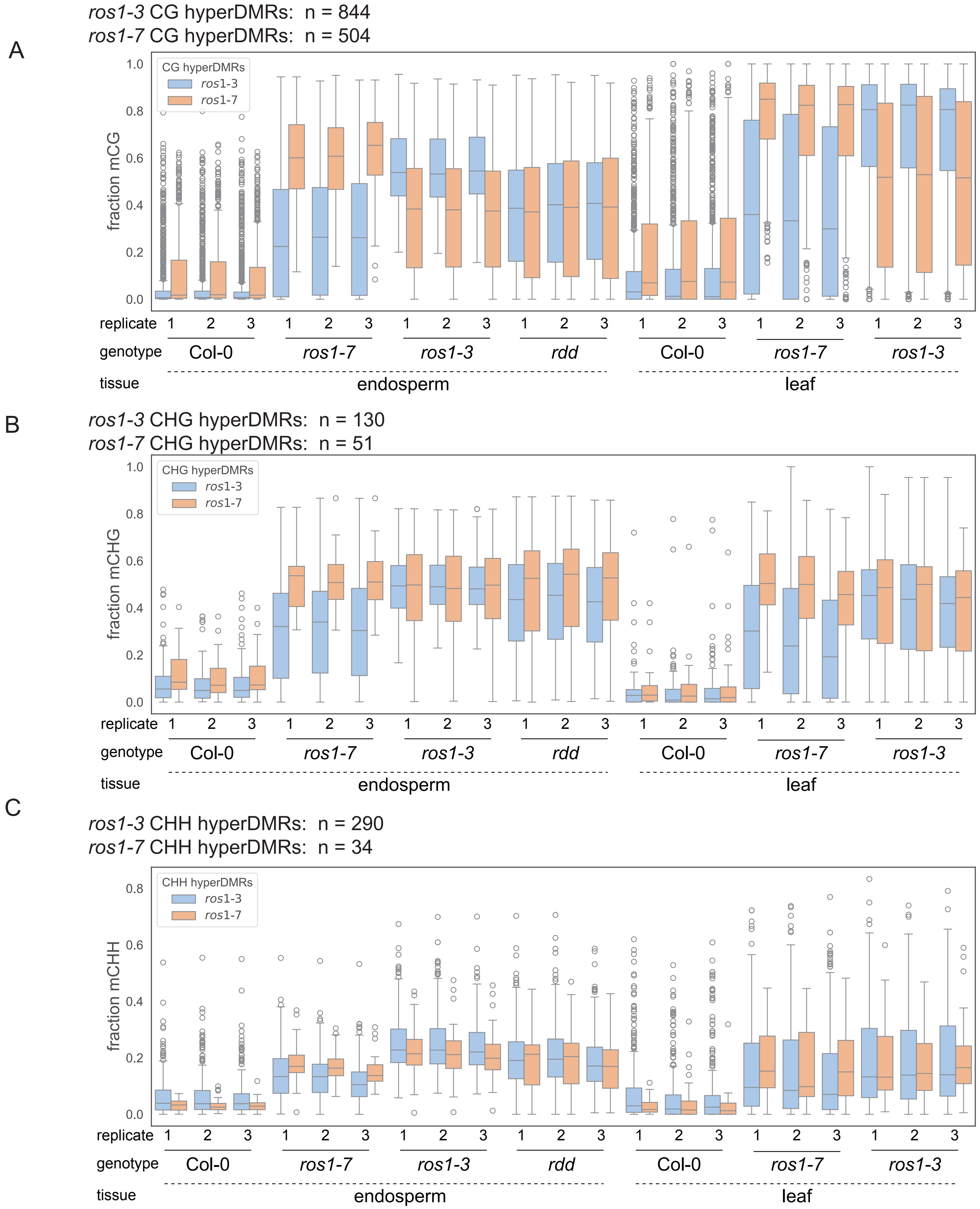

Fig. S4: Average DNA methylation levels of *ros1* endosperm hyperDMRs across biological replicates and genotypes. Weighted average of A) mCG, B) mCHG, and C) mCHH levels in hyper DMRs (*ros1* vs WT Col-0) identified in endosperm across endosperm and leaf tissues, including all sequenced genotypes and biological replicates. Only regions with data in all replicates were plotted. Plots are Tukey's box plots.

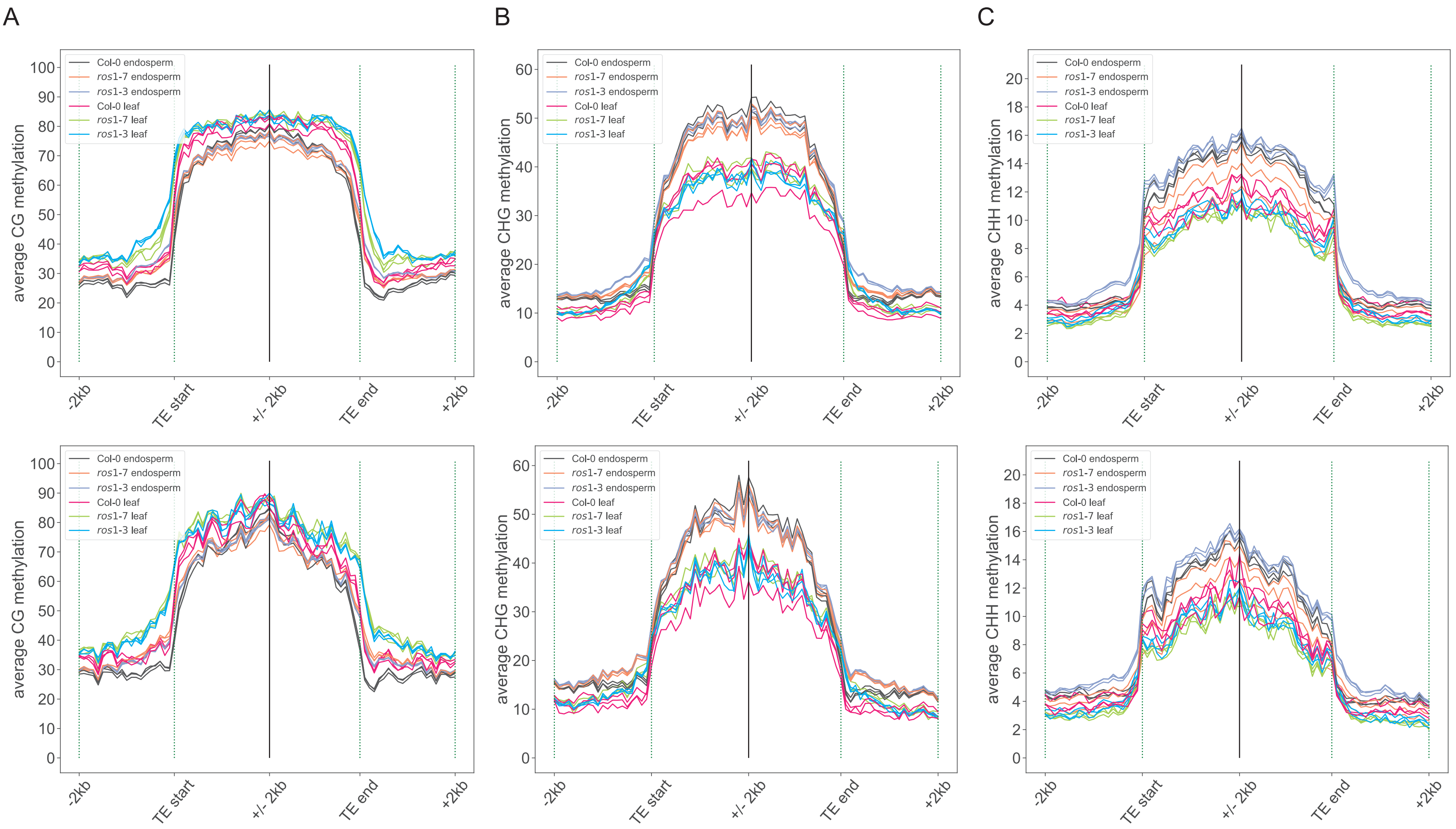

Fig. S5: DNA methylation levels at *ROS1* TEs in endosperm and leaf. Averaged levels of DNA methylation in leaf and endosperm in the A) CG, B) CHG, C) CHH sequence contexts of 100 bp windows 2 kb inside and outside of TEs within 1 kb of a *ros1-3* hyperDMR (top) or *ros1-7* hyperDMR (bottom) defined in endosperm. Biological replicates are plotted in the same color.

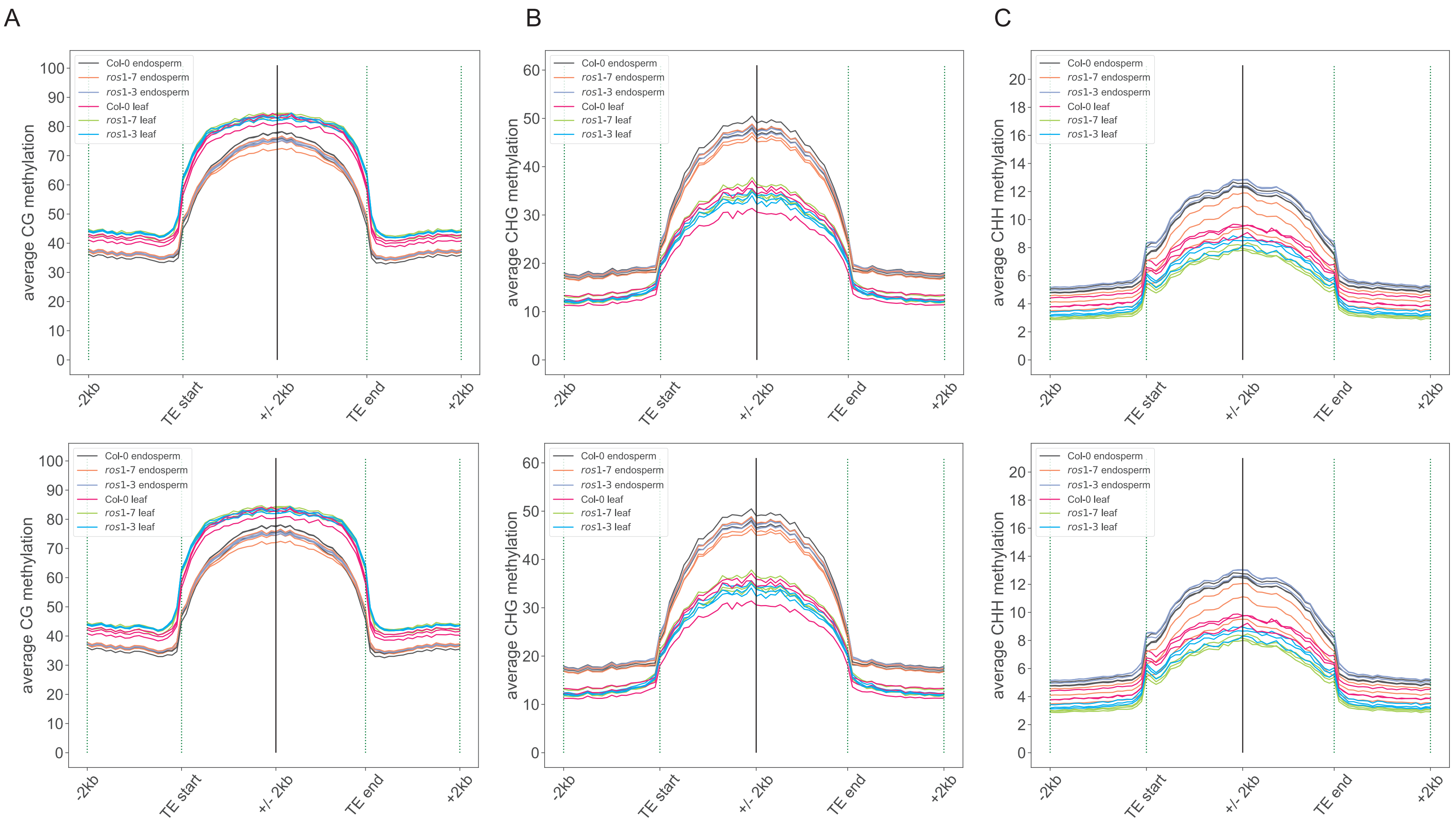

Fig. S6: DNA methylation levels at non-*ROS1* TEs in endosperm and leaf. Averaged levels of DNA methylation in leaf and endosperm in the A) CG, B) CHG, C) CHH sequence contexts of 100 bp windows 2 kb inside and outside of TEs not within 1 kb of a *ros1-3* hyperDMR (top) or *ros1-7* hyperDMR (bottom) defined in endosperm. Biological replicates are plotted in the same color.

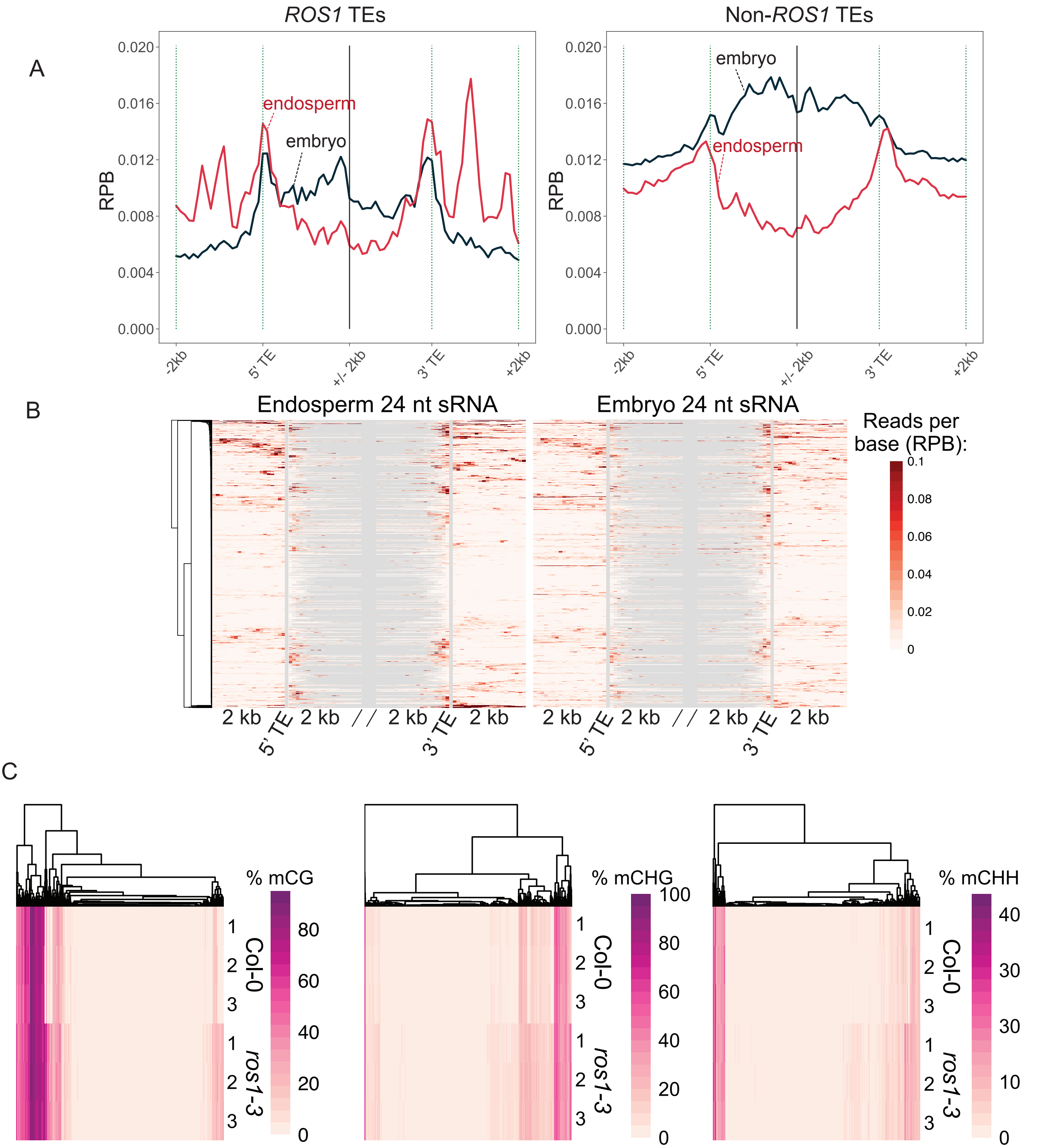

Fig. S7: ROS1 demethylates 24 nt sRNAs-producing regions in the endosperm. A) The average reads per base (RPB) of 24-nt sRNAs in the embryo and endosperm of wild-type Col-0 seeds in 100 bp windows 2 kb outside and 2 kb inside of *ROS1* TEs (defined using *ros1-3;* left) and Non-*ROS1* TEs (right). Small-RNA sequencing data from Erdmann et al 2017. B) The data underlying the metaplots in A), showing RPB for individual 100 bp windows 2 kb outside and 2 kb inside of *ROS1* TEs. Gray indicates no data. C) Weighted average DNA methylation calculated in endosperm-enriched differential sRNA regions (Erdmann et al 2017) in wild-type Col-0 and *ros1-3* endosperm. Rows missing data for any replicates were removed for these plots, n = 2481 (CG), 2598 (CHG), and 2624 (CHH) DSRs are plotted.

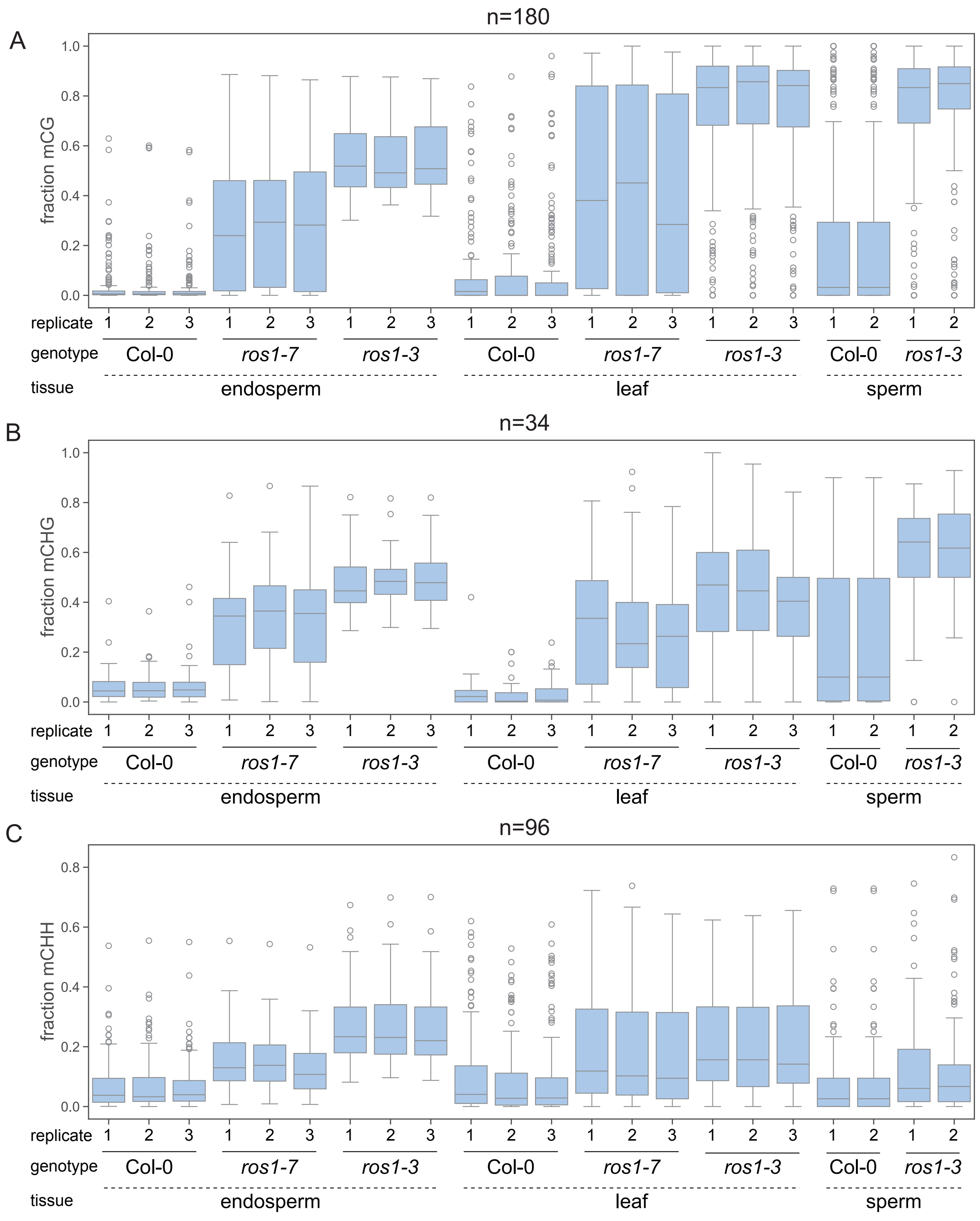

Fig. S8: CG hypermethylation is limited in *ros1* mutant endosperm relative to leaf or sperm. Weighted average of A) mCG, B) mCHG, and C) mCHH levels in *ros1-3* hyperDMRs across all replicates and genotypes in sequenced endosperm and leaf samples. Only regions with data in all replicates are plotted. Plots are Tukey's box plots.

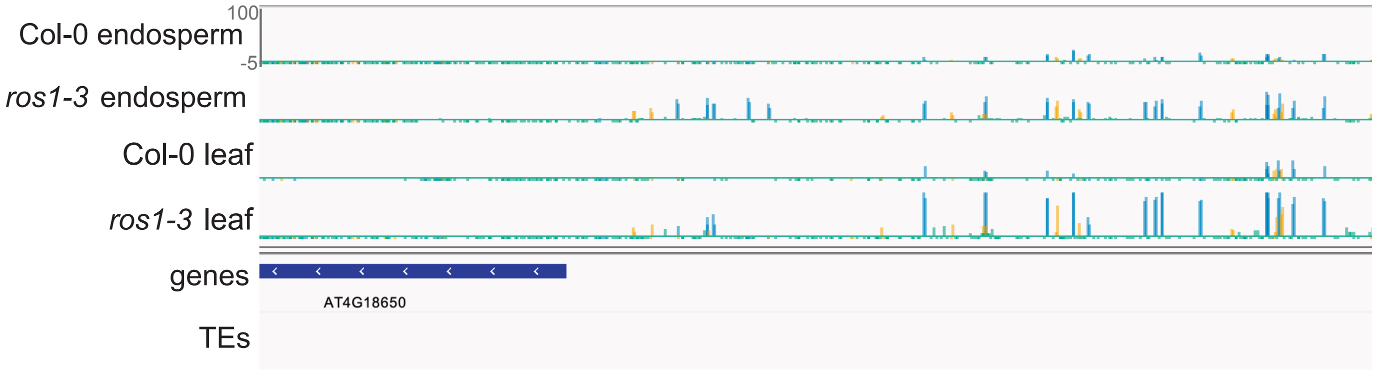

Fig. S9: Genome browser displaying 5mC in Col-0 and *ros1-3* leaf and endosperm at the *DOGL4* locus (AT4G18650). Blue=mCG, gold=mCHG, green=mCHH.

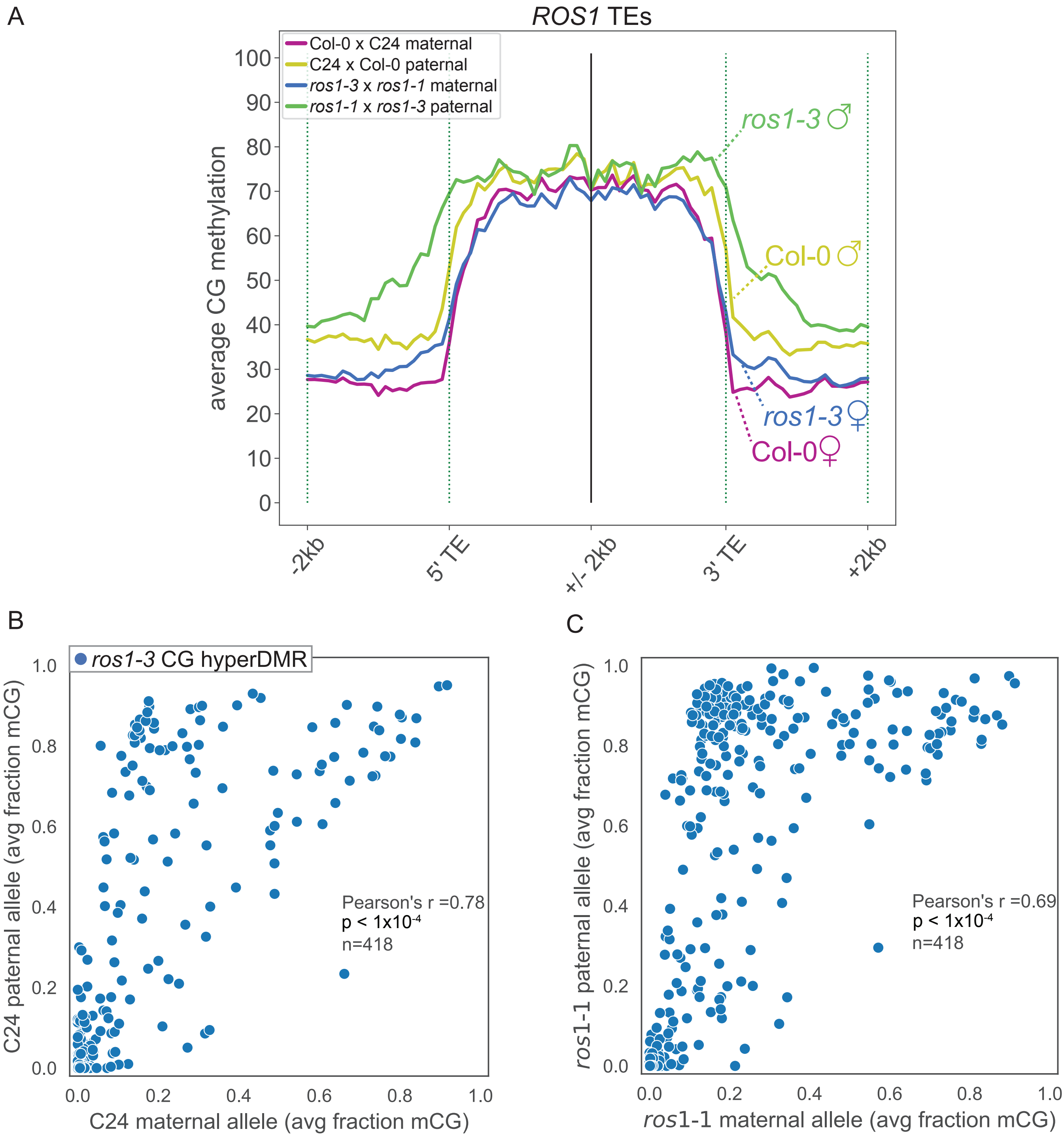

Fig. S10: CG hypermethylation in *ros1* mutant endosperm is biased for the paternal allele. A) CG methylation of Col-0 and *ros1-3* maternal and paternal genomes from selected biological replicates in F1 endosperm across 100 bp windows, 2 kb inside and 2 kb outside each end of *ROS1* TEs (defined using *ros1-3*). B) As in Figure 4A-B, weighted sum mCG levels for *ros1-3* CG hyperDMRs on maternal and paternal genomes in C24xCol (C24 maternal) and ColxC24 (C24 paternal) and in C) *ros1-1* x *ros1-3* (*ros1-1* maternal) and *ros1-3* x *ros1-1* (*ros1-1* paternal).

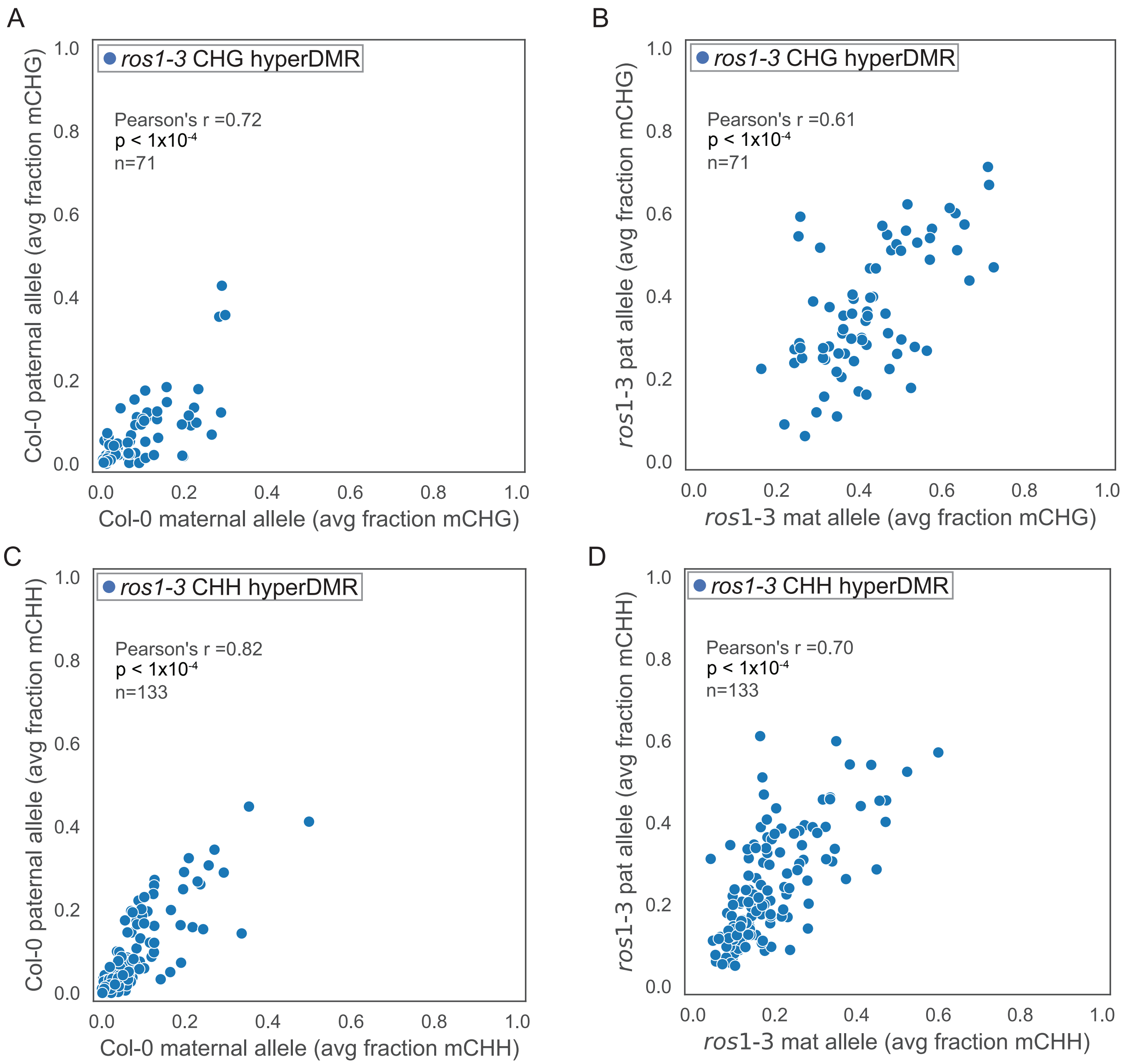

Fig. S11: Non-CG hypermethylation in *ros1* mutant endosperm is biallelic. A-B) As in Figure 4A-B, but summed mCHG levels for *ros1-3* CHG hyperDMRs. C-D) As in Figure 4A-B, but summed mCHH levels for *ros1-3* CHH hyperDMRs.

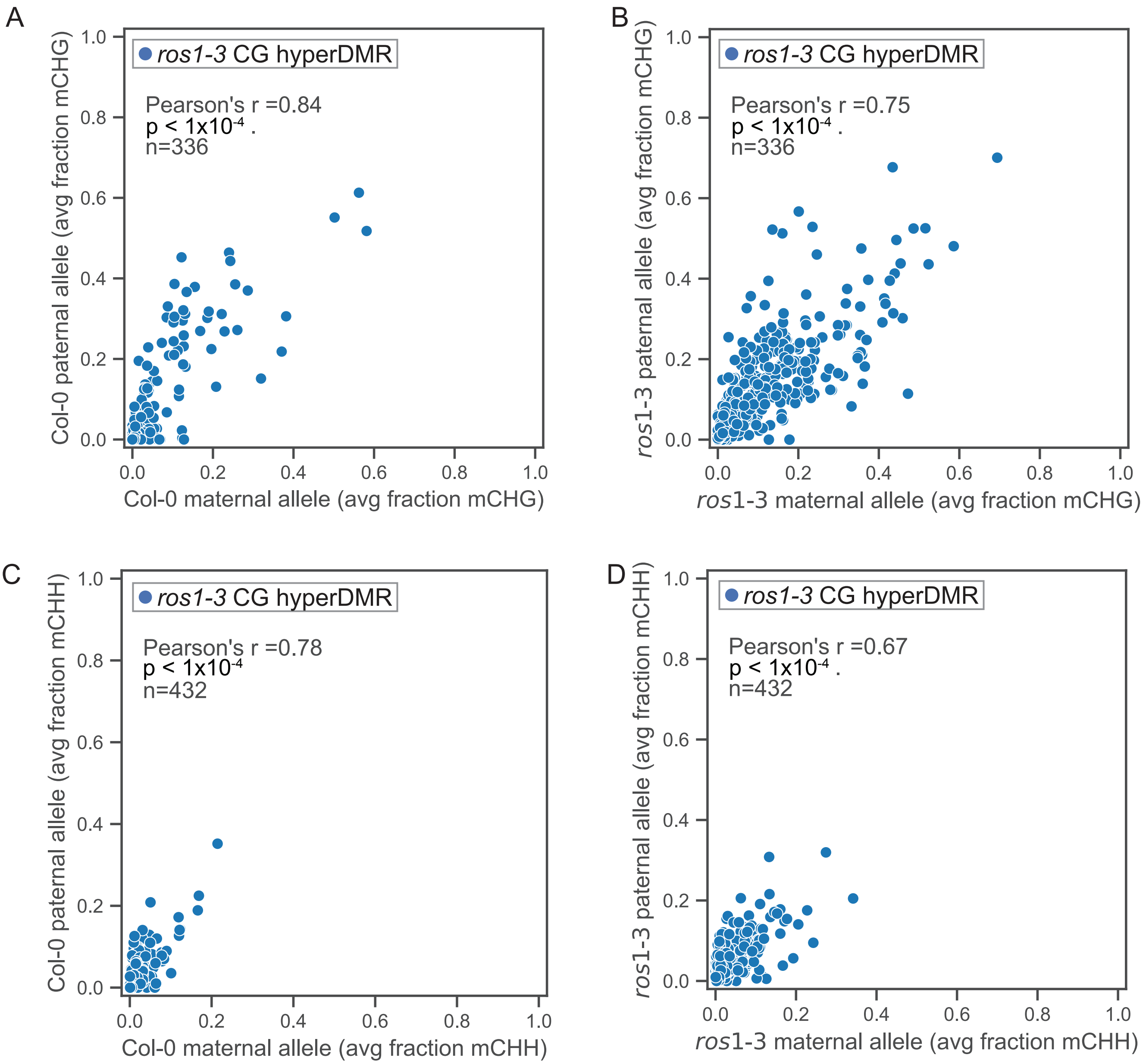

Fig. S12: Non-CG hypermethylation in endosperm *ros1-3* CG hyperDMRs is biallelic. A-B) As in Figure 4A-B, but summed mCHG levels for *ros1-3* CG hyperDMRs. C-D) As in Figure 4A-B, but summed mCHH levels for *ros1-3* CG hyperDMRs.

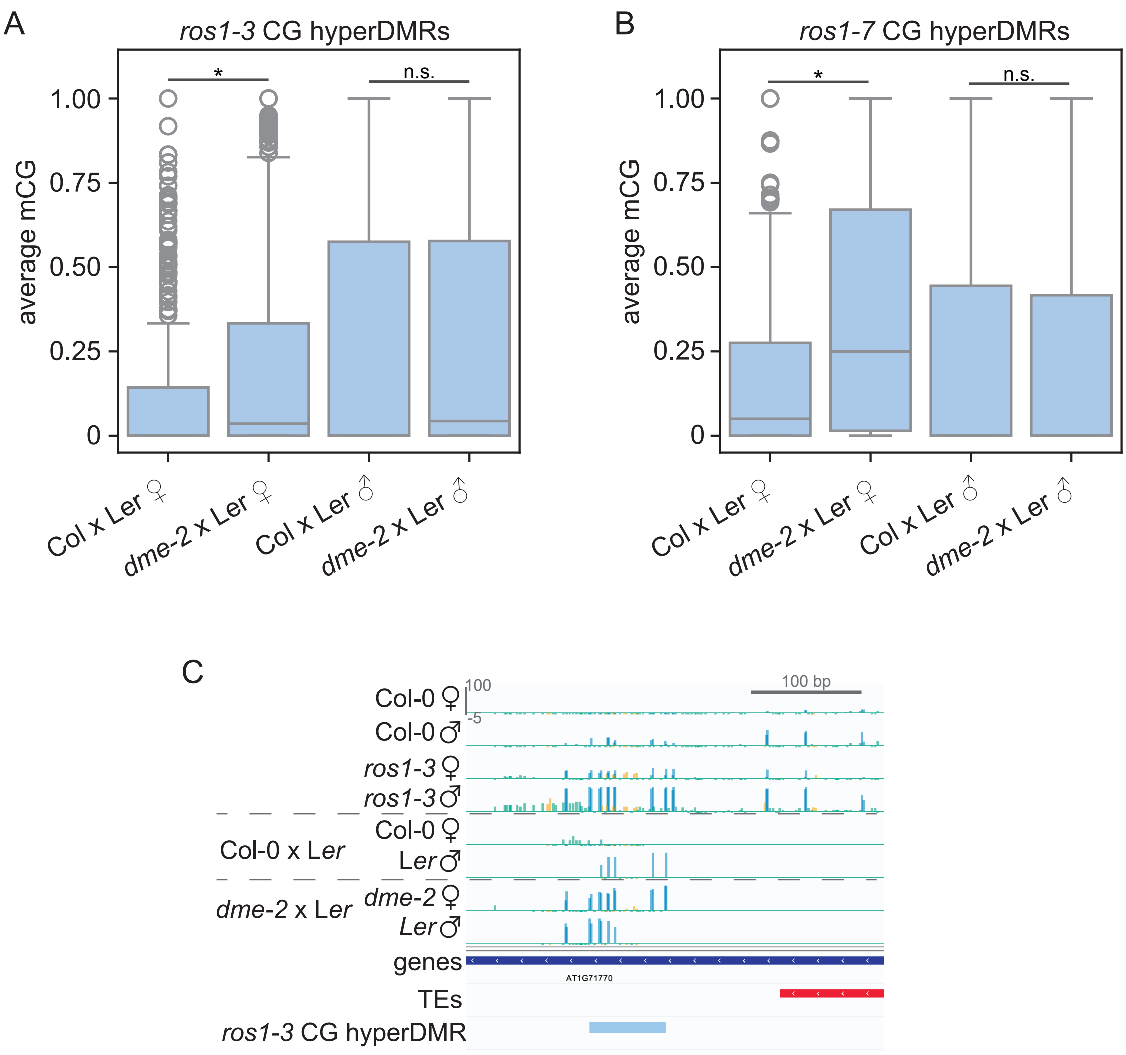

Fig. S13: *DME* demethylates the maternal allele at some *ROS1* target regions. A-B) Weighted average of fraction mCG at maternal alleles of A) *ros1-3* CG hyperDMRs (n = 385 (mat alleles), 139 (pat alleles) regions with sufficient data to plot) and B) *ros1-7* CG hyperDMRs (n = 239 (mat alleles), 85 (pat alleles) regions with sufficient data to plot) in F1 endosperm. C) A genome browser example showing maternal allele hypermethylation at a *ros1-3* CG hyperDMR when *dme-2* is inherited maternally. *dme-2* data is from Ibarra *et al*., 2012. * indicates Bonferonni-corrected p-value < 0.05, Welch's unpaired t-test. Box plots are Tukey's box plots.

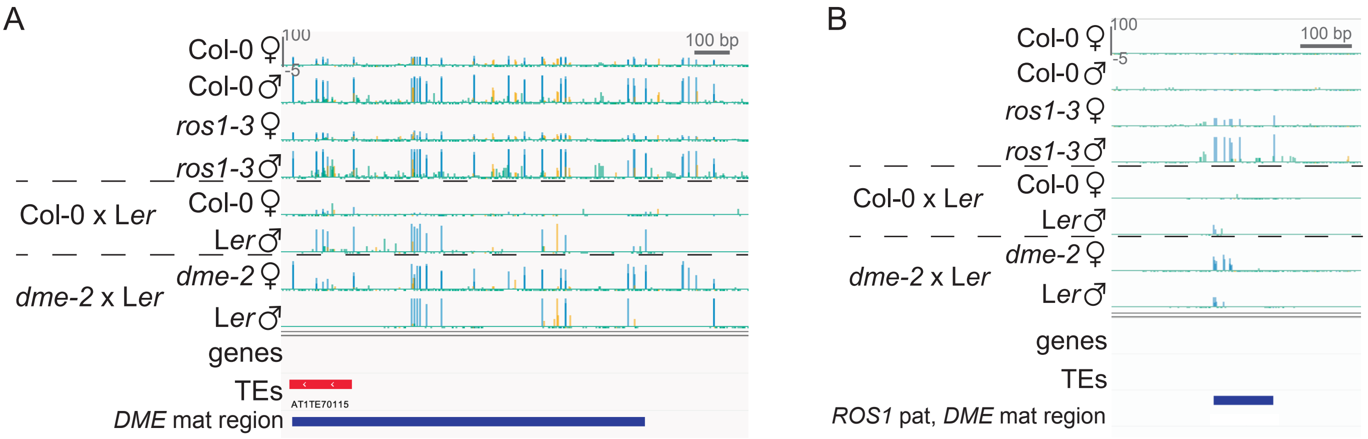

Fig. S14: DME prevents maternal allele hypermethylation at identified *DME* maternal regions and *ROS1* paternal, *DME* maternal regions. Genome browsing examples of a A) *DME* maternal region and B) a *ROS1* paternal, *DME* maternal region. The top four rows show Col-0 and *ros1-3* DNA methylation levels at this region on the maternal and paternal alleles. The bottom four rows show DNA methylation levels on the maternal and paternal alleles of Col x *Ler* F1 endosperm and *dme-2* x *Ler* F1 endosperm, from Ibarra *et al.,* 2012.

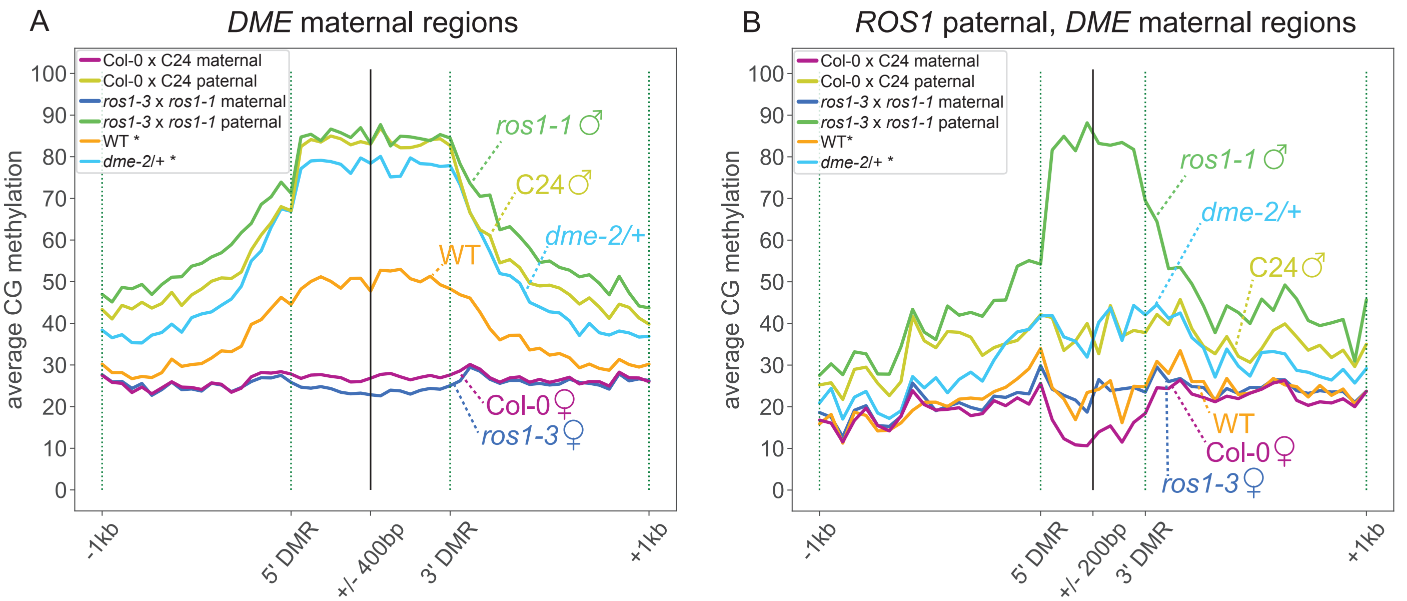

Fig. S15: DNA methylation levels in reciprocal cross direction at maternally-hypomethylated regions. Averaged levels of mCG in reciprocally crossed Col-0 x C24 and *ros1-3* x *ros1-1* endosperm data as well as wild-type and *dme* data from Hsieh *et al*., 2009 in 50 bp windows 200 or 400 bp inside and 1 kb outside of A) *DME* maternal regions and B) *ROS1* paternal, *DME* maternal regions. Hsieh *et al* data (indicated by * in legend) is not allelic, meaning maternal and paternal alleles cannot be distinguished.

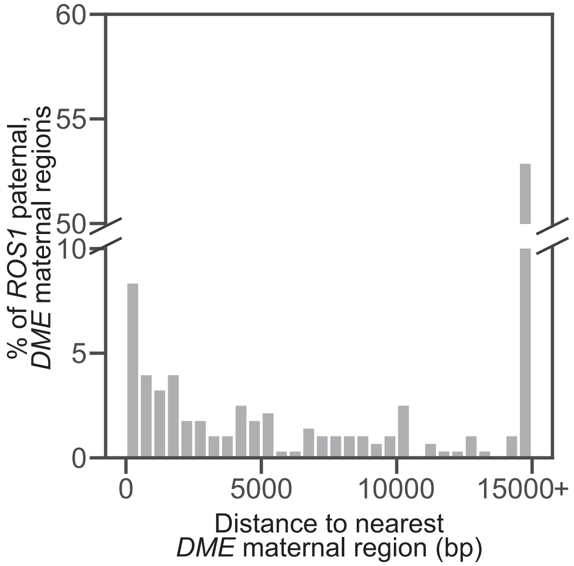

Fig. S16: Most *ROS1* paternal, *DME* maternal regions occupy distinct regions of the genome from *DME* only regions. The distance between *ROS1* paternal, *DME* maternal (n=274) to the closest *DME* maternal region, plotted as percentage of regions in 500 bp bins.

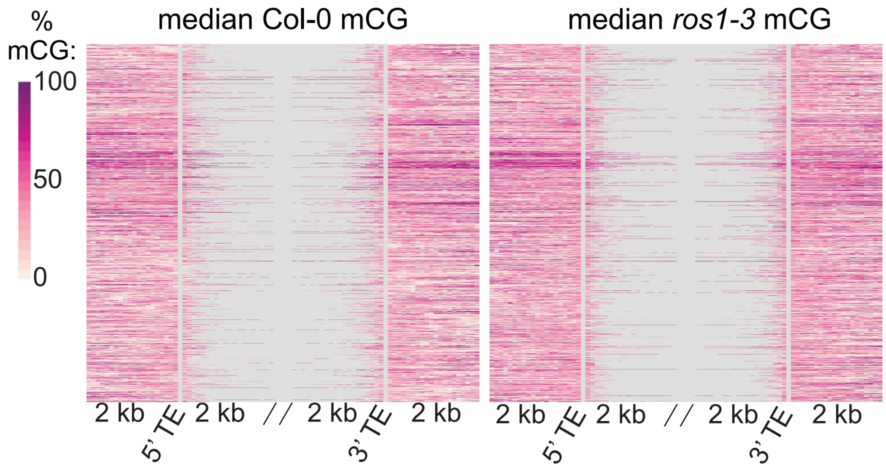

Fig. S17: *ROS1* prevents hypermethylation in flanking regions of some DME-targeted TEs. Underlying data for Fig. 7C, the median Col-0 mCG value (left) and the median *ros1-3* mCG value (right) across 100 bp windows, 2 kb inside and 2 kb outside each end of TEs within 1 kb or intersecting a *DME* maternal region. TEs are ordered the same as in Fig. 7C.

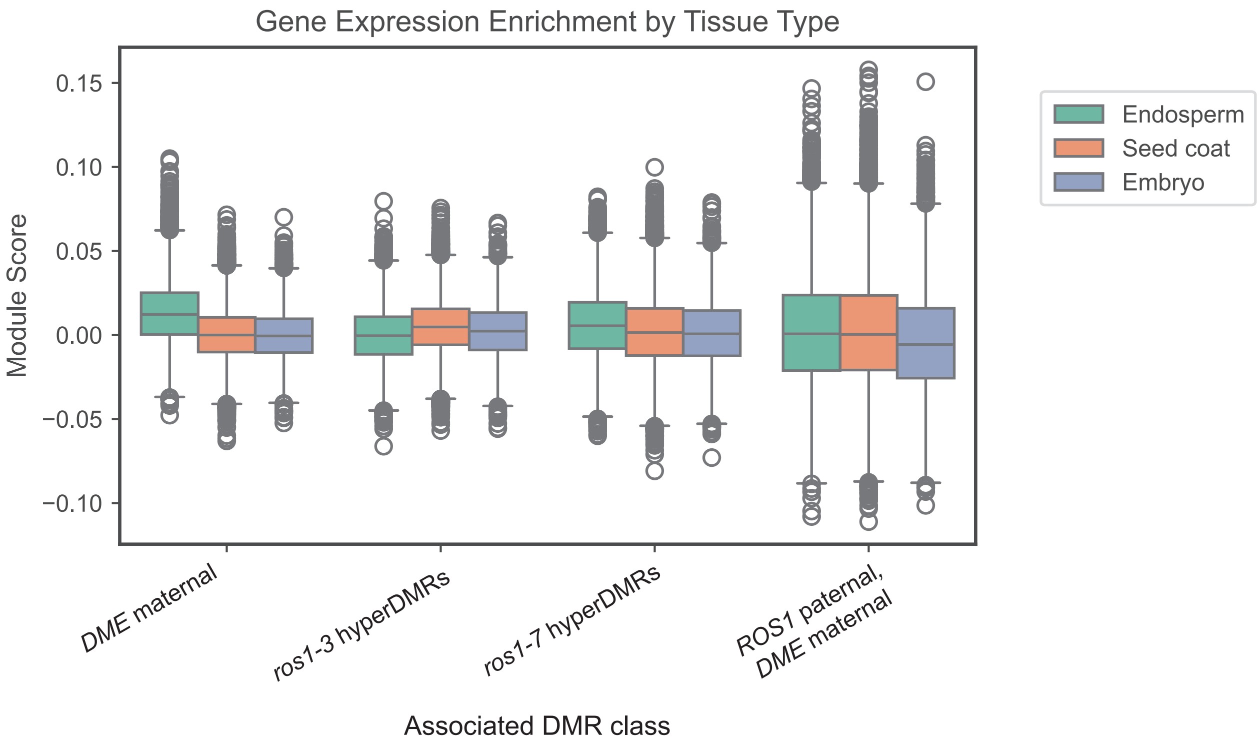

Fig. S18: Expression of genes adjacent to endosperm DMRs in wild-type seed. Relative enrichment/depletion of genes within 1 kb or intersecting an endosperm DMR across seed tissue types. Data from pooled single-nucleus RNA sequencing data of 3, 5, and 7 DAP Col-0 seedfrom Martin *et al*., 2025.

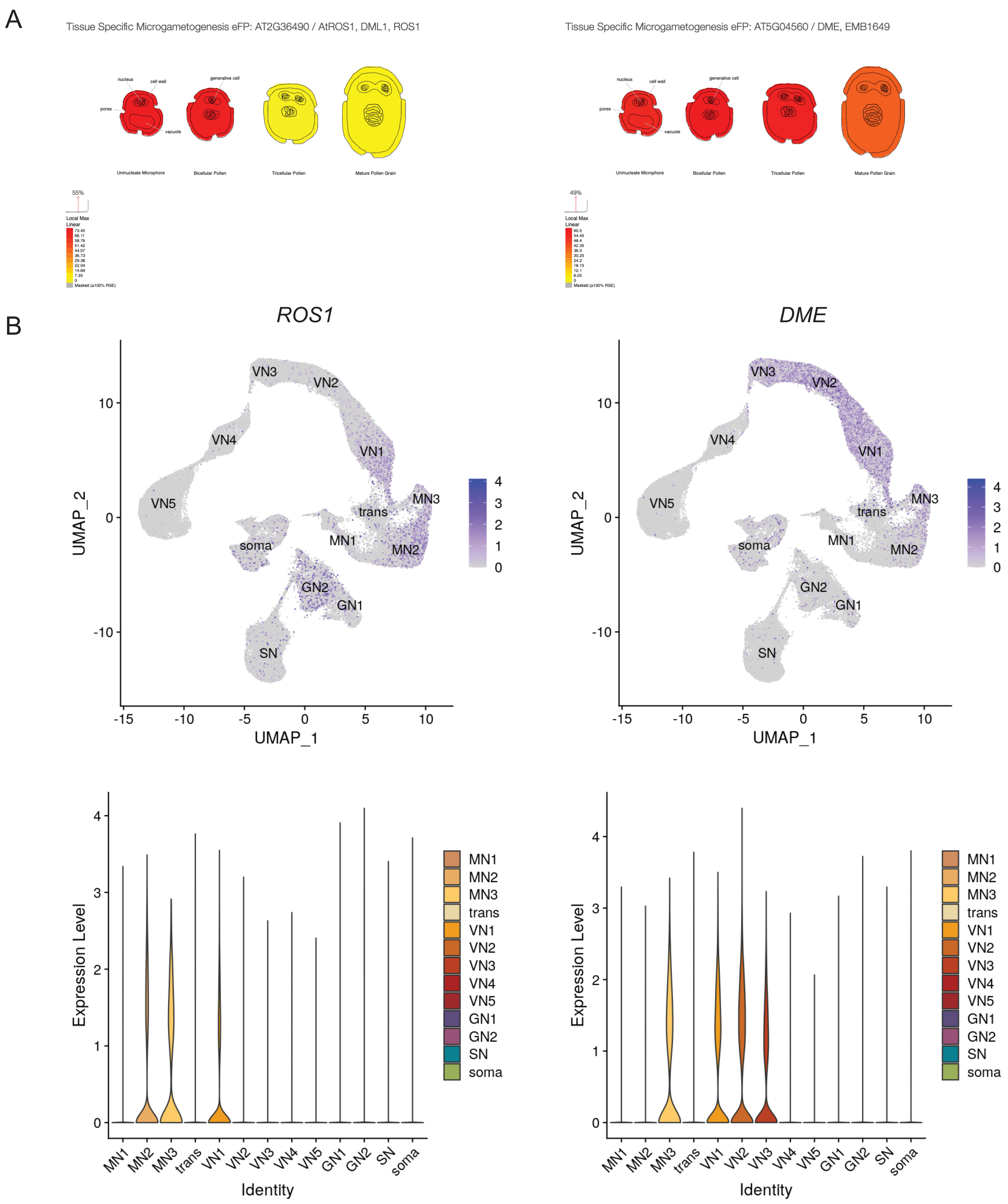

Fig. S19: *ROS1* and *DME* expression in pollen progenitor cells. A) *ROS1* (left) and *DME* (right) expression in pollen. Plots from https://bar.utoronto.ca/eplant/, using data generated in Honys & Twell, 2004. B) *ROS1* (left) and *DME* (right) expression in single-nucleus pollen gene expression data from Ichino et al, 2022.

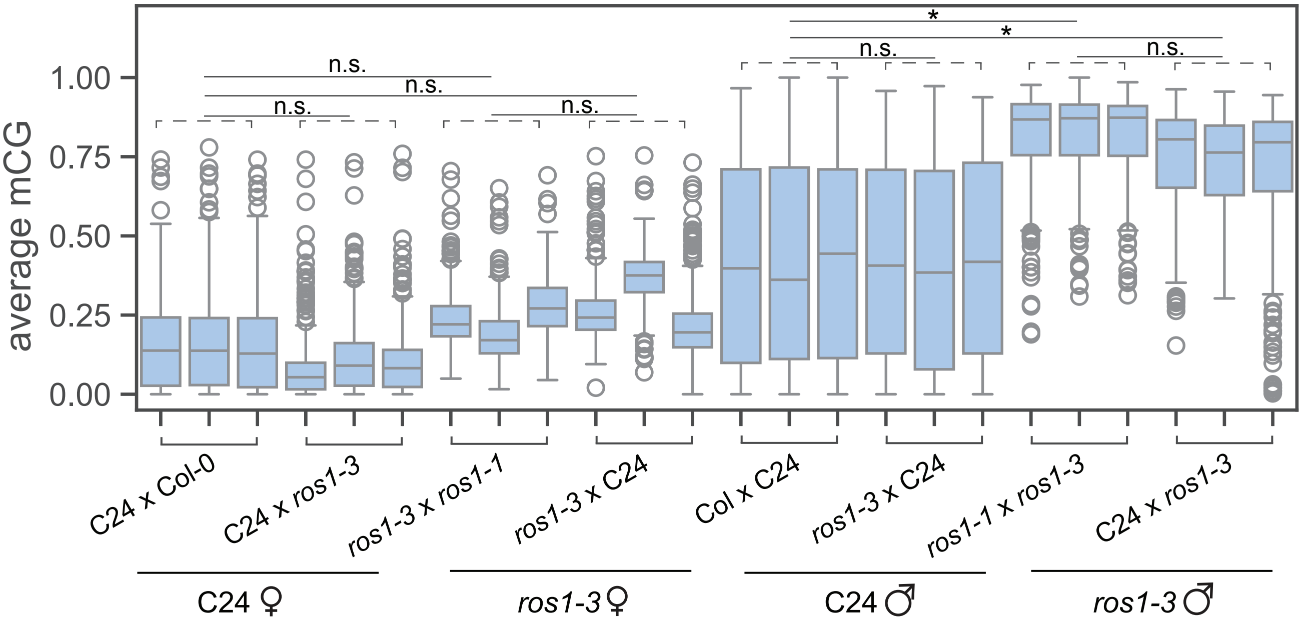

Fig. S20: Paternal allele hypermethylation of *ROS1* paternal, *DME* maternal regions is inherited paternally. Weighted average of mCG levels of C24 wild-type and *ros1-3*-inherited alleles of *ROS1* paternal, *DME* maternal regions in endosperm data. (n = 262 regions with sufficient data to plot). Female parent in cross listed first. * indicates Bonferroni corrected p-value < 0.05, Welch's unpaired t-test performed between replicate averages. Plots are Tukey's box plots.

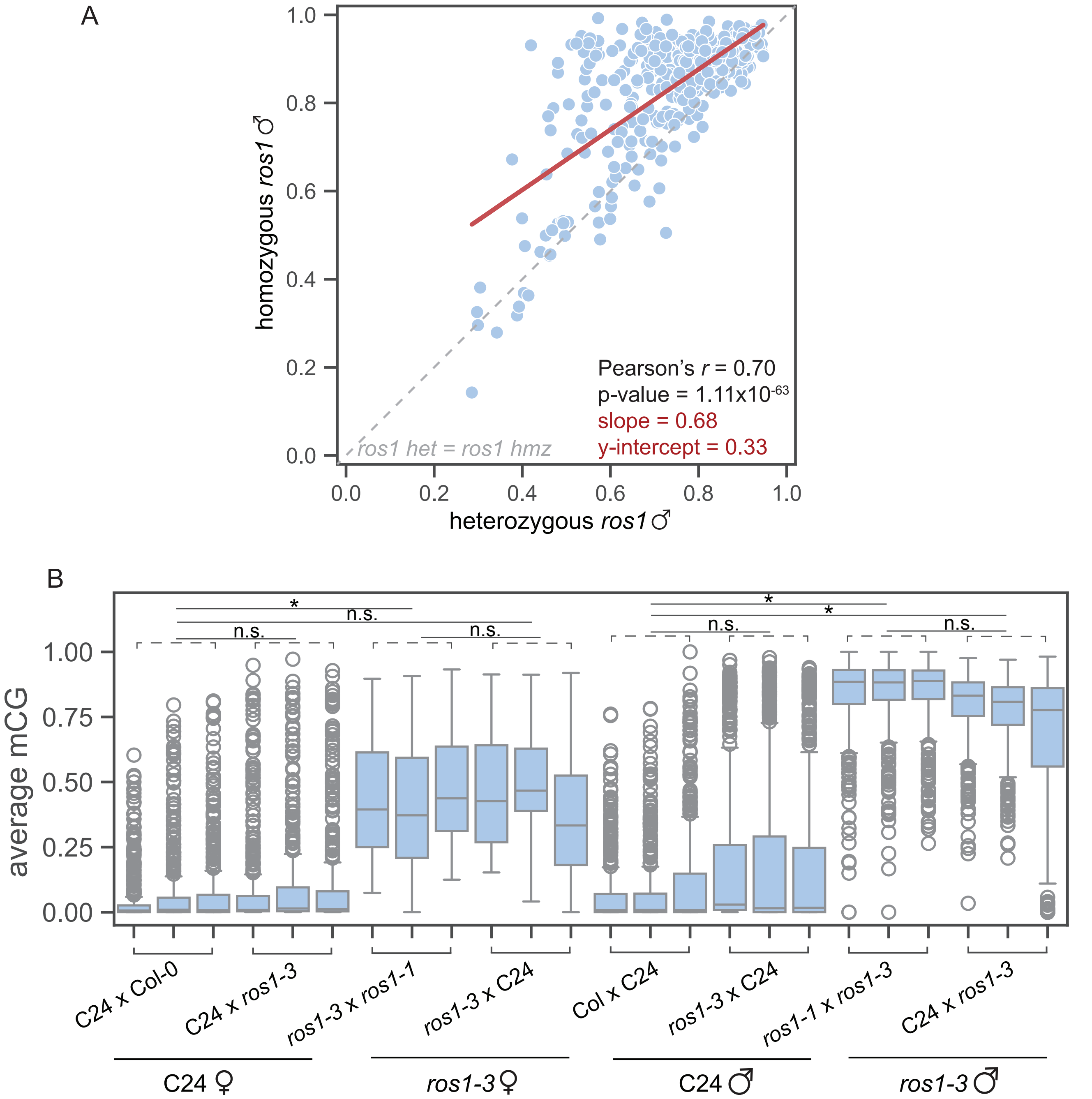

Fig. S21: Paternal hypermethylation of *ros1-3* CG hyperDMRs in the endosperm is inherited paternally. A) Weighted average mCG of the paternal allele of *ros1-3* CG hyperDMRs (n = 418). Values are averaged across biological replicates of *ros1-1* x *ros1-3* F1 endosperm (y-axis) or C24 x *ros1-3* F1 heterozygous endosperm (x-axis). Paternal allele methylation of *ros1-3* CG hyperDMRs was correlated between the homozygous and heterozygous F1 endosperm, where the *ros1-3* allele was inherited paternally (Pearson's r = 0.70, p-value = 1.11×10^{-63}). Weighted average of mCG levels of C24 wild-type and *ros1-3* inherited alleles of *ros1-3* CG hyperDMRs in endosperm data (n = 418 regions with sufficient data to plot). Female parent in cross listed first. * indicates Bonferroni corrected p-value < 0.05, Welch's unpaired t-test performed between replicate averages. Plots are Tukey's box plots.

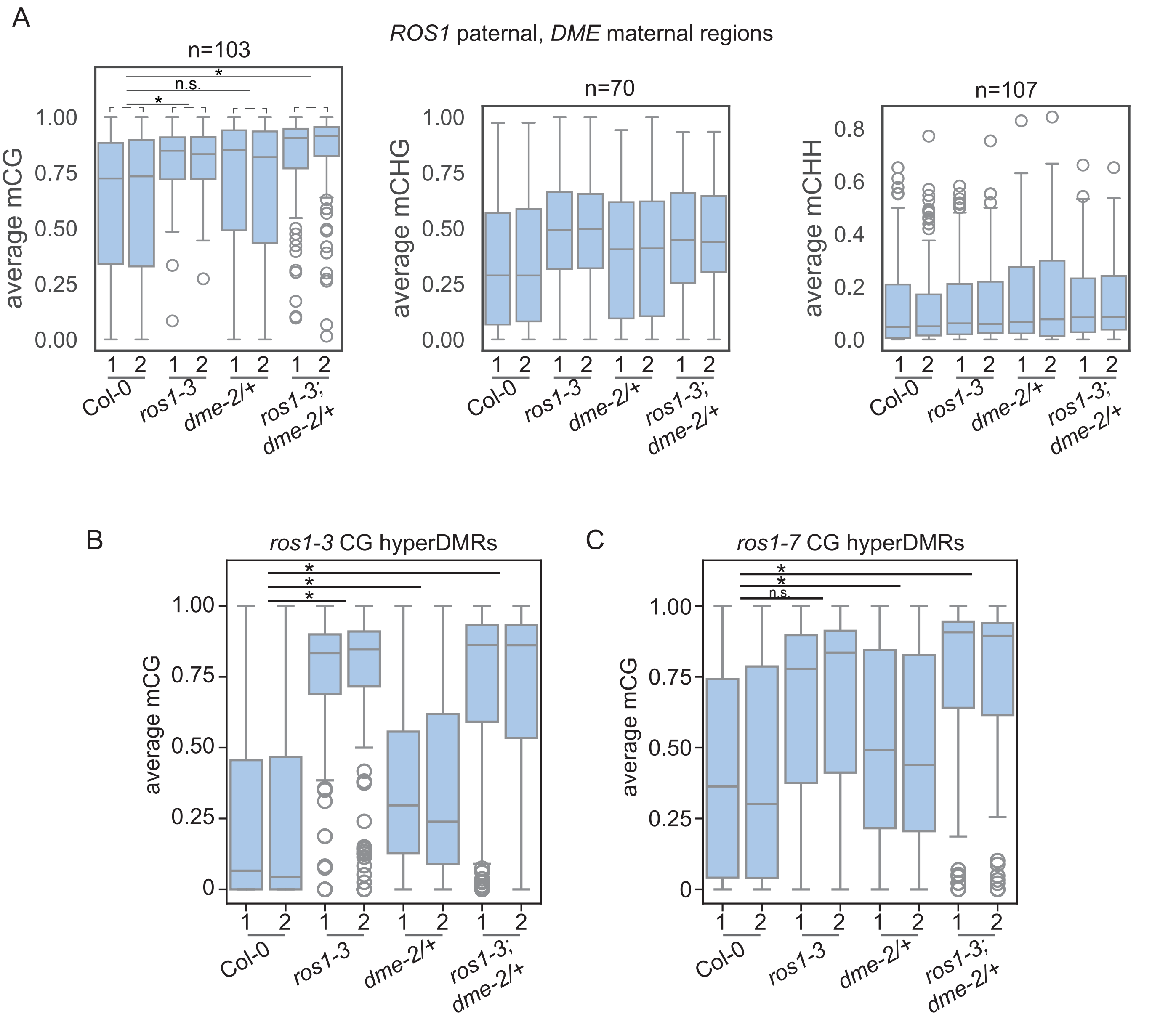

Fig. S22: A limited effect of DME is observed at endosperm ROS1 targets in sperm. A-C) Weighted average of A) mCG, B) mCHG, C) mCHH levels in *ROS1* paternal, *DME* maternal regions in Col-0, *ros1-3, dme-2/+,* and *ros1-3;dme-2/+* sperm data from Khouider et al 2021. D-E) Weighted average of mCG levels in D) *ros1-3* CG hyperDMRs (n=134) and E) *ros1-7* CG hyperDMRs (n= 80) in Col-0, *ros1-3, dme-2/+,* and *ros1-3;dme-2/+* sperm data from Khouider et al 2021. * indicates Bonferroni corrected p-value < 0.05, Welch's unpaired t-test performed between replicate averages. Plots are Tukey's box plots. Only DMRs with sufficient data in all replicates were included in analysis.
