# Supplementary material for: The 5-methylcytosine DNA glycosylase ROS1 prevents paternal genome hypermethylation in Arabidopsis endosperm
Source: Genome Biol. 2025 Sep 18;26:286. doi: 10.1186/s13059-025-03745-w (PMC12445040; doi:10.1186/s13059-025-03745-w)
Supplement: Supplementary file 7 — Additional file 7: Review history. [file 13059_2025_3745_MOESM7_ESM.docx]

Review History

**First round of review**

**Reviewer 1**

**Comments to author:**

Review Commons *Report 1*. https://doi.org/10.15252/rc.2025293060

**Reviewer 2**

**Comments to author:**

Review Commons *Report 2*. https://doi.org/10.15252/rc.2025290399

**Reviewer 3**

**Comments to author:**

Review Commons *Report 3*. https://doi.org/10.15252/rc.2025017643

**Authors Response:**

Review Commons *Response*. [**https://doi.org/10.15252/rc.2025700224**](https://doi.org/10.15252/rc.2025700224)

**Second round of review**

**Reviewer 1**

The authors sufficiently addressed my comments. I have no further comments.

**Reviewer 2**

In their revised manuscript. Hemenway and Gehring have answered my questions and addressed my concerns.

I would like to suggest making the main text more quantitative though. There are multiple places where including numbers would improve it. For example, in the section on siRNAs and DMRs there are non-quantitative descriptions such as "relatively few", "at a small magnitude", and "at some regions". Can the data be interpreted with sufficient confidence to make these quantitative? For example, could "relatively few" be replaced with something like "n% of loci accounted for N% of the increase"? Or could "a small magnitude" be replaced by an "increase from an average of X% to an average of Y%? Or could "at some regions" be replace by "at Z% of regions"? Changes like these would make the manuscript stronger and more interpretable.

And a minor point for possible clarification in the Abstract: Does "prevents hypermethylation" mean the same thing as removes methylation? The wording is a bit confusing to me, so I wonder if I am missing something significant. The rest of that sentence also seems a bit convoluted.

**Reviewer 3**

Correct DNA methylation patterning is particularly important for seed development in plants. In normal seeds, the endosperm is hypomethylated specifically on the maternal alleles due to activity of the demethylase DME in the central cell prior to fertilization. Loss of maternal DME expression causes endosperm defects and seed abortion. The function of DME is well studied, due to its dramatic phenotype. However, there are three other homologs in the same gene family in Arabidopsis: ROS1, DML2, and DML3, whose function during endosperm development is not known. In this work, Hemenway and Gehring investigate the role of these three demethylases in endosperm. They find that while DML2 and DML3 have little effect, ROS1 is important for demethylation of the paternal allele in endosperm at a subset of sites targeted by DME. While DME activity usually leads to demethylation of only the maternal alleles in endosperm, which is associated with epigenetic phenomena like genomic imprinting, ROS1 seems to counter that at some sites by also demethylating the paternal allele, bringing both maternal and paternal methylation to similar levels. Overall, this is an interesting finding that is well supported by the data, although I would appreciate it if the authors could address the points below.

If ROS1 demethylates in sperm prior to fertilization (and assuming both sperm are identical and fertilize egg/central cell at random), then regions demethylated in endosperm due to ROS1 should also be somewhat demethylated in embryo - could the authors check published embryo DNA methylation datasets to see if the ROS1 paternal DME maternal regions are mildly demethylated in wild-type embryo as well?

How to reconcile Fig. 1A and 1C? 1A shows rdd more severe, 1C shows rdd equivalent to (or maybe less severe than) ros1-3. Where are the cytosines that gain mC in rdd but not ros1 in 1A, relative to the DMRs identified in C? Are the mCs sparsely distributed such that they don't add up to a whole DMR?

Line 276 - "The maternal allele of ros1-3 and ros1-7 CG hyperDMRs were hypermethylated in dme endosperm."
What about the paternal allele of dme mutants? There is evidence that DME causes demethylation of the central cell in pollen, what about the sperm cells?

I would have appreciated more characterization of the two different types of DMRs - where do the DME maternal regions and ROS1 paternal and DME maternal regions occur in the genome (chromosome arms or pericentromere)? For the ones in genes or promoters, is there anything affected genes have in common? Maybe they're all highly expressed in the endosperm or share similar functions? For the regions not associated with any genes, are there any common sequence motifs or other sequence-level similarities? Are they associated with specific chromatin states?

Line 346 - "More than 86% of DME maternal regions and 75% of ROS1 paternal DME maternal regions are associated with TEs only, and are not within 1kb of or intersecting a gene." This does not agree with the figure - nearly half of both sets of regions are within 1kb of both a gene and a TE.

Loss of ROS1 doesn't cause any obvious phenotypes, and in this work, the DNA methylation gain and affected regions in endosperm are modest. But previous work on ros1 has shown that defects can accumulate over several generations. The experiment is probably too much to ask for, but can the authors speculate whether the methylation changes seen here in endosperm similarly become more severe after a few generations propagation of homozygous ros1, and whether that might eventually affect seed development?

It would be interesting to check other published pollen RNA-seq data to see where and when DME and ROS1 are expressed in pollen, since there have been quite a few published lately. Several are cell type specific or test multiple timepoints during pollen development. Offhand, I would suggest looking at Khouider et al. 2021 Nat Commun., as well as the single-nucleus RNA data from Ichino et al. 2022 Cell Rep.

Are there changes in DME or DML2/3 expression in ros1, or changes in ROS1 expression in dme endosperm?

Minor comments:
- Several typos in line 64
- Fig. S1 legend has panel D mislabeled as C
- Line 386 - typo: bicellular

**Authors Response**

**Point-by-point responses to the reviewers’ comments:**

Reviewer #1: The authors sufficiently addressed my comments. I have no further comments.

Reviewer #2: In their revised manuscript. Hemenway and Gehring have answered my questions and addressed my concerns.

I would like to suggest making the main text more quantitative though. There are multiple places where including numbers would improve it. For example, in the section on siRNAs and DMRs there are non-quantitative descriptions such as "relatively few", "at a small magnitude", and "at some regions". Can the data be interpreted with sufficient confidence to make these quantitative? For example, could "relatively few" be replaced with something like "n% of loci accounted for N% of the increase"? Or could "a small magnitude" be replaced by an "increase from an average of X% to an average of Y%? Or could "at some regions" be replace by "at Z% of regions"? Changes like these would make the manuscript stronger and more interpretable.

*Response: Thank you for the suggestion. We have added additional quantification to the siRNA section and other sections where possible. To quantify the endosperm enrichment of 24-nt small RNAs relative to embryo sRNAs at ROS1 TEs, defined as TEs within 1 kb or intersecting a ros1-3 vs Col-0 hyperDMR, we first summed the sRNA level across windows in flanking regions of ROS1 TEs in both tissues. Then, we identified TEs with a summed level of endosperm sRNAs relative to the embryo greater than 1 SD from the mean difference across all ROS1 TEs. 25 ROS1 TEs meet this threshold. This indicates the data are very skewed towards no difference between endosperm and embryo levels, and these 25 TEs have extremely high levels of 24 nt sRNAs in the endosperm. This information is now included in the text and in Supplemental Table 3.*

And a minor point for possible clarification in the Abstract: Does "prevents hypermethylation" mean the same thing as removes methylation? The wording is a bit confusing to me, so I wonder if I am missing something significant. The rest of that sentence also seems a bit convoluted.

*Response: This is something we have discussed frequently, and we appreciate your insight. We have used the phrasing “prevents hypermethylation” to refer to the molecular phenotype in the endosperm associated with a loss of ROS1 function. We are cautious to not make reference to the protein activity of ROS1 as it relates to demethylation in the endosperm, as the results of our study can largely be explained by inheritance of a ros1 effect through the male gametes (Figure 8).*

Reviewer #3:
Correct DNA methylation patterning is particularly important for seed development in plants. In normal seeds, the endosperm is hypomethylated specifically on the maternal alleles due to activity of the demethylase DME in the central cell prior to fertilization. Loss of maternal DME expression causes endosperm defects and seed abortion. The function of DME is well studied, due to its dramatic phenotype. However, there are three other homologs in the same gene family in Arabidopsis: ROS1, DML2, and DML3, whose function during endosperm development is not known. In this work, Hemenway and Gehring investigate the role of these three demethylases in endosperm. They find that while DML2 and DML3 have little effect, ROS1 is important for demethylation of the paternal allele in endosperm at a subset of sites targeted by DME. While DME activity usually leads to demethylation of only the maternal alleles in endosperm, which is associated with epigenetic phenomena like genomic
imprinting, ROS1 seems to counter that at some sites by also demethylating the paternal allele, bringing both maternal and paternal methylation to similar levels. Overall, this is an interesting finding that is well supported by the data, although I would appreciate it if the authors could address the points below.

*Response: Thank you for your positive comments. We have addressed all points below.*

If ROS1 demethylates in sperm prior to fertilization (and assuming both sperm are identical and fertilize egg/central cell at random), then regions demethylated in endosperm due to ROS1 should also be somewhat demethylated in embryo - could the authors check published embryo DNA methylation datasets to see if the ROS1 paternal DME maternal regions are mildly demethylated in wild-type embryo as well?

*Response: To address this point, we analyzed Col-0 embryo methylation data published in Williams et al, Plant Cell, 2022. Note that this data was from mature embryos, a later developmental stage than what we profiled for endosperm. We compared methylation in ROS1 endosperm DMRs in endosperm, leaf, sperm and embryo. Unfortunately only 65 regions have data available to plot in both the sperm and embryo. We have not included this analysis in the paper as it is preliminary and lacks sufficient coverage. Additionally, the expectations for these* *regions at embryos is unclear – it is known that the methylation is reinforced/gained in embryos throughout development; thus one might expect that even if a hypomethylated region was inherited from the sperm it would not persist in the embryo.*

How to reconcile Fig. 1A and 1C? 1A shows rdd more severe, 1C shows rdd equivalent to (or maybe less severe than) ros1-3. Where are the cytosines that gain mC in rdd but not ros1 in 1A, relative to the DMRs identified in C? Are the mCs sparsely distributed such that they don't add up to a whole DMR?

*Response: This apparent discrepancy has remained a bit of a puzzle to us. We have considered several possible explanations. First, less of the genome could be assayed for methylation differences in rdd compared to ros1. This is because we removed from consideration regions of the genome that were introgressed from Ws (the ecoptype origin of the ros1-3 and dml2-1 alleles) into Col. There are 50 DMRs (merged, all contexts) that are called in ros1-3 vs wild-type that reside in regions that could not be assessed for DMRs in rdd. However, these regions are included in our calculation of total genome methylation. This may explain some of the difference, but certainly not all. We also investigated whether the rdd biological replicates were more variable, and thus the pipeline we chose for DMR calling, which incorporates a dispersion statistic to account for biological replicates, would reduce the number of DMRs called. In support of this idea, we find that across all CG sites, ros1-3 endosperm has a mean dispersion value of 0.0092, while the mean dispersion value across CG sites in rdd is 0.011. We think this slight difference in variability between biological replicates also underlies the fewer DMR calls for rdd. In general, we are cautious about relying on the presence or absence of a DMR in a single sample throughout the paper, and are careful to validate our observations using multiple alleles, ecotypes, and analyses.*

Line 276 - "The maternal allele of ros1-3 and ros1-7 CG hyperDMRs were hypermethylated in dme endosperm."
What about the paternal allele of dme mutants? There is evidence that DME causes demethylation of the central cell in pollen, what about the sperm cells?

*Response: The dme data we have utilized from Ibarra et al 2012 is endosperm from a cross between a dme heterozygous maternal parent and a wild-type paternal parent. We have clarified this line to better represent this. We have included analysis of methylation of the paternal wild-type allele from dme/+ heterozygous endosperm in Supplemental Fig 13. It is almost identical to the paternal allele in wild-type endosperm. Please note that fewer regions are included in the paternal plots due to coverage differences.*

*We also used data from Khouider et al 2021 to look at possible effects of DME at ros1 hyperDMRs in sperm cells derived from a dme heterozygote. We find that at ros1-3, ros1-7 and ros1-1 CG hyperDMRs, there is a slight difference between Col-0 and dme/+ sperm, but not as severe as the hypermethylation observed in ros1-3 sperm for all sets of endosperm hyperDMRs. This analysis suggests that there is limited redundancy of ROS1 and DME at regions demethylated by ROS1 in the male germline, with the caveat that the dme sperm methylation data analyzed is a mixture of WT and dme mutant sperm. Thank you for this suggestion, this is an interesting result and is now included in Supplemental Figure 22 and discussed in the text.*


I would have appreciated more characterization of the two different types of DMRs - where do the DME maternal regions and ROS1 paternal and DME maternal regions occur in the genome (chromosome arms or pericentromere)? For the ones in genes or promoters, is there anything affected genes have in common? Maybe they're all highly expressed in the endosperm or share similar functions? For the regions not associated with any genes, are there any common sequence motifs or other sequence-level similarities? Are they associated with specific chromatin states?

*Response: To further characterize our DMRs, we first performed the analysis you suggest, and plotted the distribution of DMRs over chromosomes. ROS1 DMRs, both ros1-3 hyperDMRs and ROS1/DME DMRs (ROS1 paternal, DME maternal) are evenly distributed across the chromosomes. DME DMRs, however, are denser in pericentromeric regions, where there are more transposable elements. This is consistent with previous results showing that endosperm vs embryo hypoDMRs, indicative of DME activity in the central cell, match the distribution of transposable elements in the genome (Gehring et al, Science 2009). We have included this analysis in Figure 7A.*

*This initial observation, and your suggestion, led us to further investigate the chromatin state at defined demethylase targets. We utilized information on the 9 chromatin states defined by Sequeira-Mendes et al, Plant Cell 2014 in seedling tissue (note that it is unknown whether these same chromatin states are present in reproductive or endosperm tissue). Consistent with the distribution of DMRs, we find that, in seedling tissue, DME regions are enriched in H3K9me2 marked heterochromatin, with 66% of DME maternal regions being found in chromatin state 8. The largest fraction of chromatin states represented in both ros1-3 and ros1-7 hyperDMRs is also state 8, but there is more representation of states 5 and 4, which are marked by H3K27me3. The largest fraction of ROS1 paternal, DME maternal regions, 48%, are found in chromatin state 4, indicating these shared targets of ROS1 and DME are mostly found in intergenic regions marked by H3K27me3. These data are included Figure 7F.*

*We further focused our investigation on H3K27me3 in the endosperm, which has been shown to be relevant for imprinted status of some genes. For example, many paternally expressed imprinted genes are associated with a H3K27me3 and a DME-dependent hypoDMR on the maternal allele. The relationship between DNA methylation and H3K27me3 is not straightforward, though some studies report an anticorrelation between DNA methylation and H3K27me3 in the endosperm (Weinhofer et al, PLoS Genet. 2010). Using data from H3K27me3 profiling in Col x Ler endosperm at 4 days after pollination (Zheng and Gehring, Plant Reproduction, 2019), we evaluated if ROS1 and DME regions are coincident with H3K27me3 peaks in the endosperm We find that 42% of endosperm ros1 hyperDMRs are associated with an H3K27me3 peak in endosperm tissue. By contrast, ~20% of DME regions are associated with an H3K27me3 peak in endosperm. ROS1/DME regions are in between these values, with ~29% of regions associated with an H3K27me3 peak in endosperm. These results are consistent with the chromosomal localization and chromatin state of these regions, as H3K27me3 is relatively depleted from pericentromeric regions. These results are presented in the text.*

*To probe possible transcriptional effects of demethylation in seeds, we assessed expression in wild-type Col-0 from a recent single-nucleus RNA-seq dataset from our lab (Martin et al, bioRxiv 2025). We considered the expression of genes found within 1 kb or intersecting DME maternal regions and ROS1 paternal, DME maternal regions. We also evaluated the genes closest to ros1-3 and ros1-7 hyperDMRs (ROS1 genes), where we show hypermethylation is paternally-biased. We found that the expression of genes near DME regions are enriched in the endosperm compared to the embryo or seed coat, but that the expression of ROS1 and ROS1/DME genes are not enriched in endosperm relative to embryo or seed coat. We have added this analysis to Supplemental Figure 18. It is possible that in demethylase mutant endosperm additional gene expression patterns would appear, which would be an interesting direction for future study.*

*Given the previously identified relationship between ROS1 and seed germination, published in Zhu et al, Proc Natl Acad Sci USA 2018, we asked if genes with enriched expression during seed germination, profiled in Kawakatsu et al, Genome Biology 2017, are enriched among genes near ros1 hyperDMRs and ROS1/DME regions, and if this is different from those near DME maternal regions. We observed no such enrichment. However, this does not exclude that there might be individual target genes, in addition to DOGL4 that are involved in seed germination.*

Line 346 - "More than 86% of DME maternal regions and 75% of ROS1 paternal DME maternal regions are associated with TEs only, and are not within 1kb of or intersecting a gene." This does not agree with the figure - nearly half of both sets of regions are within 1kb of both a gene and a TE.

*Response: Thank you for pointing this out, we have corrected this statement.*

Loss of ROS1 doesn't cause any obvious phenotypes, and in this work, the DNA methylation gain and affected regions in endosperm are modest. But previous work on ros1 has shown that defects can accumulate over several generations. The experiment is probably too much to ask for, but can the authors speculate whether the methylation changes seen here in endosperm similarly become more severe after a few generations propagation of homozygous ros1, and whether that might eventually affect seed development?

*Response: All of the mutant lines we are working with have been maintained in the lab for multiple generations, with the exception of ros1-1 in the C24 background, which was recently obtained from the stock center. ros1-3 and ros1-7 have been propagated by self-fertilization for a minimum 5 generations. Answering the reviewer’s questions experimentally would require isolating a “fresh” ros1 mutation, or backcrossing current mutants to wild-type. While possible, we do not think this would add substantially to the current work, nor would speculation be fruitful at this stage.*

It would be interesting to check other published pollen RNA-seq data to see where and when DME and ROS1 are expressed in pollen, since there have been quite a few published lately. Several are cell type specific or test multiple timepoints during pollen development. Offhand, I would suggest looking at Khouider et al. 2021 Nat Commun., as well as the single-nucleus RNA data from Ichino et al. 2022 Cell Rep.

*Response: We have added data on ROS1 and DME expression in pollen from Ichino et al to Supplemental Figure 19. Consistent with the earlier Honys and Twell data, and as described in the Ichino paper, ROS1 is expressed primarily in the late microspore nucleus and to a lesser extent in the early vegetative nucleus of bicellular pollen. DME is expressed in the late microspore and most strongly in the early vegetative nucleus. See also published Figure S5 in Ichino et al.*

Are there changes in DME or DML2/3 expression in ros1, or changes in ROS1 expression in dme endosperm?

*Response: Hsieh et al, Proc Natl Acad Sci USA, 2011 indicated that ROS1 is expressed more highly in dme+/- x Ler F1 endosperm than in Col x Ler F1 endosperm. However, this was one of many gene expression changes in dme. We do not think a differential expression analysis using these data before has been reported, but examining genes that the authors analyzed for parent of origin-specific expression (n= 10,755), we calculate 7870 have more reads in dme/+, though we have not performed a true differential expression analysis or statistics. We do not think this analysis is relevant for the current study. In somatic tissue, it has been reported that 3,874 genes are significantly up-regulated in dme-2 mutant, compared to 1,448 down-regulated genes (Kim et al, PNAS 2021). To our knowledge DML2/3 and DME are not differentially expressed in a ROS1 mutant. It is possible that more maternal genome hypermethylation would be observed in F1 endosperm when a dme ros1 double mutant was used as a female in a cross, but we have not pursued this possibility as of yet.*

Minor comments:
- Several typos in line 64
- Fig. S1 legend has panel D mislabeled as C
- Line 386 - typo: bicellular

*Response: We have corrected the errors*

**Third round of review**

**Reviewer 3**

The reviewers have satisfied all of my concerns and the paper appears ready to be published.
